# Investigation of the Thermogelation of a Promising Biocompatible ABC Triblock Terpolymer and Its Comparison with Pluronic F127

**DOI:** 10.1021/acs.macromol.1c02123

**Published:** 2022-02-14

**Authors:** Anna P. Constantinou, Valeria Nele, James J. Doutch, Joana S. Correia, Roman V. Moiseev, Martina Cihova, David C. A. Gaboriau, Jonathan Krell, Vitaliy V. Khutoryanskiy, Molly M. Stevens, Theoni K. Georgiou

**Affiliations:** † Department of Materials, Imperial College London, London SW7 2AZ, UK; ‡ Department of Bioengineering, Imperial College London, London SW7 2AZ, UK; § Institute of Biomedical Engineering, Imperial College London, London SW7 2AZ, UK; ∥ ISIS Neutron and Muon Source, STFC, Rutherford Appleton Laboratory, Didcot OX11 ODE, UK; ⊥ Reading School of Pharmacy, University of Reading, Whiteknights, P.O. Box 224, Reading RG66AD, UK; # Facility for Imaging by Light Microscopy, NHLI, Imperial College London, London SW7 2AZ, UK; ∇ Department of Surgery & Cancer, Imperial College London, London SW7 2AZ, UK

## Abstract

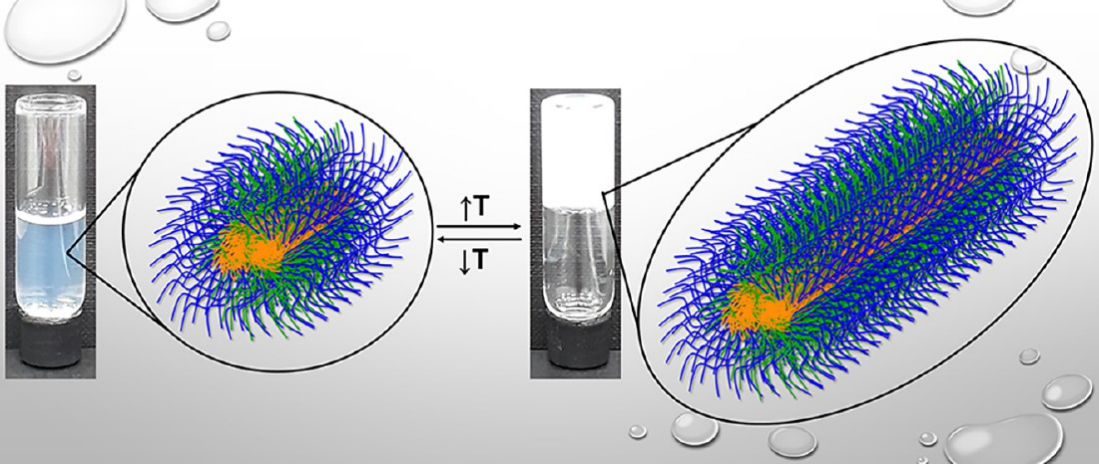

Thermoresponsive polymers with the appropriate structure form physical networks upon changes in temperature, and they find utility in formulation science, tissue engineering, and drug delivery. Here, we report a cost-effective biocompatible alternative, namely OEGMA300_15_-*b*-BuMA_26_-*b*-DEGMA_13_, which forms gels at low concentrations (as low as 2% w/w); OEGMA300, BuMA, and DEGMA stand for oligo(ethylene glycol) methyl ether methacrylate (MM = 300 g mol^–1^), *n*-butyl methacrylate, and di(ethylene glycol) methyl ether methacrylate, respectively. This polymer is investigated in depth and is compared to its commercially available counterpart, Poloxamer P407 (Pluronic F127). To elucidate the differences in their macroscale gelling behavior, we investigate their nanoscale self-assembly by means of small-angle neutron scattering and simultaneously recording their rheological properties. Two different gelation mechanisms are revealed. The triblock copolymer inherently forms elongated micelles, whose length increases by temperature to form worm-like micelles, thus promoting gelation. In contrast, Pluronic F127’s micellization is temperature-driven, and its gelation is attributed to the close packing of the micelles. The gel structure is analyzed through cryogenic scanning and transmission electron microscopy. Ex vivo gelation study upon intracameral injections demonstrates excellent potential for its application to improve drug residence in the eye.

## Introduction

Thermoreversible hydrogels (TRGs) are three-dimensional (3D) networks of noncovalently interacting self-assembled structures that can reversibly turn to the solution phase as the stimulus (i.e., temperature) is removed.^1,2^ Of particular interest are the TRGs that are formed upon a temperature increase, i.e., the ones presenting lower critical solution temperature (LCST) behavior.^1^ This fascinating class of polymers has been extensively studied by polymer scientists, engineers, and biologists over the last decades and has found utility in the biomedical sector. This is due to its reversible nature and inherent ability of physical cross-linking without additional chemicals that might induce toxicity. Specifically, LCST-type TRGs have been extensively studied as injectable gels for tissue engineering (TE)^3^ and drug/gene delivery^1^ or most recently as 3D-printable biomaterials.^4,5^ In this concept, a clear transition from solution to gel is required as the liquid phase at room temperature (r.t.) facilitates the easy mixing and loading into a syringe, while a gel is formed at body temperature (b.t.) to fulfill the desired task, i.e., tissue regeneration and/or local drug/gene delivery.

Due to its thermogelling properties and commercial availability, poloxamer 407, known as Pluronic F127, is the most used thermoresponsive polymer. Its structure is EG_99_-*b*-PG_66_-*b*-EG_99_, where EG and PG stand for ethylene glycol and propylene glycol, respectively.^1,6−8^ Pluronic F127 has reached clinical trials, which have either been completed or are in progress (NCT03400475, NCT02365389, and NCT04139018)^1,9^ as opposed to ReGel (based on EG and poly(d,l-lactide-*co*-glycolide) (PLGA-PEG-PLGA), OncoGel: ReGel/paclitaxel formulation), whose clinical trials (NCT00479765 and NCT00573131) have been discontinued.^9−11^ Both systems are in the gel state at physiological temperature, but they have a relatively high critical gelation concentration (CGC). Aqueous solutions of Pluronic F127 at concentrations of ≤12.6% w/v form highly viscous liquids at r.t., while a gel is formed at r.t. at concentrations of ∼20% w/v.^1,8^ On the other hand, ReGel’s samples are gels at r.t. at concentrations of at least 10% w/w; thus, the injection is performed at the gel phase.^10,12^

The use of a TRG with a low CGC is very advantageous as it is potentially cost-efficient and less toxic than TRGs with a high CGC. Additionally, lower polymer concentrations often correspond to improved injectability, and the TRG formed has higher porosity, which is critical for the transport of nutrients. To meet this need, several studies have reported TRGs with low CGC based on diblock copolymers such as^13−16^ (i) thermoresponsive worm gels by Armes’ group^14−16^ (at 10% w/w and 21 °C)^15^ and (ii) double hydrophobic micellar gels in water/ethanol mixtures by Hoogenboom et al. (at 1% w/w).^13^ TRGs with low CGC based on ABC triblock terpolymers have also been reported,^17−22^ including (i) a hydrogel based on a triblock terpolymer with two discrete cores by Zhou et al. (at 5% w/w and 42 °C),^21,22^ (ii) reactive oxygen species (ROS)-degradable polymer by Gupta et al. (at 2.5% w/w),^19,20^ (iii) a rhodamine-containing polymer (6% w/w and 29.5 °C),^17^ and (iv) a vinyl ether based gel (at 6% w/w and 20 °C).^18^ In addition to these, a cellulose-containing graft polymer has been reported to form hydrogels at 4.2% w/w and 37 °C.^23^ Peptide- and peptoid-based thermoresponsive systems have also been reported to form hydrogels at low concentrations.^24−28^ A hybrid triblock terpolymer formed a gel at 4% w/v,^27^ while hybrid diblock copolymers have also been reported (at 8% w/w at 35 °C^26^ and at 6% w/w at 33 °C).^25^ Notably, copolypeptoids have been reported to form hydrogels at 1% w/w,^24^ while polyisocyanipeptide-grafted oligo(ethylene glycol) formed gels at r.t. at the extremely low concentration of 0.006% w/w.^28^

Nevertheless, in preclinical and clinical trials, Pluronic F127 remains the most popular option.^1^ Due to its high CGC and high viscosity/gelation at r.t., Pluronic F127 is not suitable for applications requiring injection through narrow needles, for which sol–gel transition closer to b.t. is preferred. However, they are ideal for applications in the gel phase, e.g., epicutaneous application. Although systems with low CGC have been reported, these have not been investigated in clinical trials. Furthermore, these systems are either produced via complicated synthetic routes or via polymerization methods that are difficult to scale up, thus limiting their commercialization. In addition, some of these systems form gels at r.t., which introduces the same issues discussed previously for Pluronic F127. As such, there is a clear need for thermoresponsive polymers with low CGCs, ease of synthesis, and potential for biomedical applications.

Here, we introduce a novel thermoresponsive ABC triblock terpolymer that overcomes some of the current technological challenges of the TRG field. This terpolymer presents a low CGC (2% w/w) and reduced viscosity at r.t., and it is produced via an industrially invented and established polymerization method, namely group transfer polymerization (GTP). GTP is a cost- and time-effective method,^29−31^ allowing the ABC triblock terpolymer to be synthesized in a maximum of 45 min. In addition, GTP is well known for its quantitative yields,^29−31^ which eliminates the need of intermediate purification steps. Therefore, the upscaling to mass production of this triblock terpolymer via GTP is certainly feasible.

Our ABC triblock copolymer is based on a novel combination of methacrylate units recently patented by our group.^32,100^ More specifically, it consists of the hydrophobic *n*-butyl methacrylate (BuMA, B block) and two hydrophilic PEG-based methacrylate units, namely oligo(ethylene glycol) methyl ether methacrylate with a molar mass (MM) of 300 g mol^–1^ (OEGMA300, degree of polymerization (DP) of EG ≈ 4.5, A block) and di(ethylene glycol) methyl ether methacrylate (DEGMA, C block). This combination avoids potential toxicity issues associated with amine-based monomers.^33−37^ In addition, DEGMA shows a cloud point (CP, defined as the temperature at which the solution turns cloudy) at around 30 °C,^38−40^ while OEGMA300 presents a CP at higher temperatures (∼70 °C).^39,40^ In order to fully harness the potential of our ABC triblock copolymer, we carried out an extensive characterization of its self-assembly and rheological properties in aqueous media by using state-of-the-art techniques. Specifically, we performed visual tests, rheological measurements, dynamic light scattering (DLS), differential scanning calorimetry (DSC), cryogenic transmission and scanning electron microscopy (cryoTEM and cryoSEM), and rheology-small angle neutron scattering (rheo-SANS) to investigate its self-assembly and gelling behavior. Rheo-SANS is a powerful and non-destructive technique that enabled us to simultaneously elucidate the copolymer’s macroscopic rheological behavior and thus confirm gelation and its self-assembly at the nanoscale across a range of temperatures. To the best of our knowledge, this is the first SANS study on methacrylate ABC triblock terpolymers. We also explored the potential of our ABC triblock copolymer for ocular drug delivery, which is a well-established application for TRGs. We assessed the biocompatibility of our copolymer in arising retinal pigment epithelia (ARPE-19) cells as well as its applicability as an in vitro drug delivery depot and its injectability in an ex vivo bovine eye model. We benchmarked our in-house-synthesized polymer with Pluronic F127, which is a commercially available thermoresponsive polymer currently undergoing clinical trials.

Overall, our work introduces a new ABC triblock copolymer with good thermogelling behavior, low CGC, ease of synthesis, and applicability for ocular drug delivery, which has the potential to revolutionize the TGR field.

## Experimental Section

### Materials

All monomers used in this study were commercially available, and they were purchased from Sigma Aldrich Co., Ltd., Irvine, United Kingdom (UK): OEGMA300 (MM = 300 g mol^–1^, 95%), BuMA (99%), and DEGMA (MM = 188.22 g mol^–1^, 95%). Chemicals needed for the monomer and solvent purification, polymer synthesis, and characterization as well as ex vivo intracameral injections and toxicity studies were also purchased from Sigma Aldrich Co., Ltd., Irvine, UK: calcium hydride (CaH_2_, ≥90%, drying agent), basic aluminum oxide (Al_2_O_3_·KOH, acid remover), 2,2-diphenyl-1-picrylhydrazyl hydrate (DPPH, free-radical inhibitor), deuterated chloroform (CDCl_3_, 99.8%, NMR solvent), methyl trimethylsilyl dimethylketene acetal (MTS, 95%, GTP initiator), tablets of phosphate-buffered saline (PBS), Pluronic F127, Pluronic F68, sodium fluorescein (SF), tetrahydrofuran anhydrous (THF, polymerization solvent, inhibitor-free, ≥ 99.9%), polyethyleneimine (PEI), and dimethylsulfoxide (DMSO). The catalyst for synthesis, namely tetrabutylammonium bibenzoate (TBABB), required the use of tetrabutylammonium hydroxide (40% in water) and benzoic acid purchased from Acros Organics (UK distributor: Fisher Scientific UK Ltd., Loughborough, UK). Fisher Scientific UK Ltd. was also the provider of Dulbecco’s modified Eagle medium (DMEM), Phalloidin-647 dye, ProLong Diamond mounting medium, Hoechst dye, phosphate-buffered saline (PBS) solution (10× solution, solvent for phase diagrams), PBS tablets, *n*-hexane (precipitation solvent), and polytetrafluoroethylene (PTFE) hydrophilic syringe filters (0.45 μm pore size, 25 mm diameter). Acros Organics was the provider of deuterium oxide (D_2_O, 99.8%D). THF (HPLC grade, not stabilized, mobile phase in chromatography) was purchased from VWR International Ltd., Lutterworth, UK. Poly(methyl methacrylate) (PMMA) standard samples (MM = 2, 4, 8, 20, 50, and 100 kDa), used as calibrants for the gel permeation chromatography (GPC) system, were purchased from Fluka, Sigma Aldrich Co., Ltd., Irvine, UK.

### Purification of Starting Materials

The low-MM monomers (BuMA and DEGMA) were purified in four steps: (i) passing twice through basic alumina, (ii) addition of DPPH, (iii) addition of the desiccant CaH_2_, and (iv) vacuum distillation. The high-MM monomer, OEGMA300, was purified in two steps as a solution in THF (50% v/v) (steps i and iii). Direct filtration into the polymerization flask was performed using PTFE filters. The initiator (MTS) was vacuum-distilled prior to use, while the catalyst (TBABB) was previously synthesized following a standard protocol^41^ and purified through recrystallization. All the glassware used for the vacuum distillations and polymerization were dried overnight at 140 °C.

### Polymer Synthesis and Purification

The in-house-synthesized triblock terpolymer, OEGMA300_15_-*b*-BuMA_26_-*b*-DEGMA_13_ (experimental structure), was synthesized via sequential GTP. Specifically, around 10 mg of the catalyst, TBABB, was added in a one-neck round bottom flask, which was then sealed with a septum and purged with argon to ensure complete substitution of the atmosphere by inert argon gas. Subsequently, freshly purified THF (59 mL) was syringed into the flask, followed by the addition of the MTS (0.6 mL, 0.5 g, 3 mmol). Three monomer additions were carried out as follows: (i) OEGMA300 solution in THF (17 mL, 8.9 g, 30 mmol), (ii) BuMA (8.7 mL, 7.8 g, 55 mmol), and (iii) DEGMA (5.4 mL, 5.5 g, 29 mmol). The exothermic GTP after each monomer addition was confirmed by using a temperature controller attached to the flask. After each polymerization step was completed, two aliquots (0.1 mL each) were withdrawn for GPC and ^1^H NMR analysis. The polymer was purified and recovered via precipitation in cold *n*-hexane. The purification was completed by vacuum-drying for 10 days at r.t..

### Gel Permeation Chromatography

For this analysis, an Agilent SEC GPC system was used, which was purchased from Agilent Technologies UK Ltd., Shropshire, UK. This system is equipped with an Agilent guard column (PL1110–1520, PLgel Mixed, dimensions: 50 mm × 7.5 mm, particle size: 5 μm) and a PSS (stands for polymer standard service) SDV analytical linear M column (SDA083005LIM, dimensions: 300 mm × 8.00 mm, particle size: 5 μm, separation range: 0.1–1000 kg mol^–1^). The system also consists of a “1260 Iso” isocratic pump, which operates at a 1 mL min^–1^ working flow rate and an Agilent 1250 refractive index (RI) detector. The system was calibrated using six PMMA standard samples of MM values equal to 2, 4, 8, 20, 50, and 100 kg mol^–1^. The GPC solvent pumped through the system was THF. All the samples were prepared using the eluent solvent, and they were filtered into GPC vials using PTFE filters (0.45 μm of pores diameter) before analysis. The GPC data were analyzed using a PSS software (WinGPC UniChrom 8.2 software from PSS-Polymer). The experimental MMs were compared to the theoretical ones (MM_theoretical_), which were calculated using by using the equation ; MM_i_ and DP_i_ denote the MM and degree of polymerization of each different monomer, while 100 g mol^–1^ corresponds to the part of the initiator that stays on the polymer chain after the completion of the dissociative GTP.
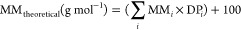


### Nuclear Magnetic Resonance Spectroscopy

The NMR samples were prepared by dissolving the dried polymer samples obtained during GTP in deuterated chloroform. The solutions were analyzed by using a 400 MHz Avance Bruker NMR spectrometer from Bruker (Bruker, UK Ltd., Coventry, UK). The data were analyzed using MestReNova software (version 11.0.0-17609, 2016 Mestrelab Research S.L.). The experimental weight percentages were calculated by using the following peaks: (i) for OEGMA300 and DEGMA, which are both EG-based methacrylate units, the distinctive peak of their methoxy group at the end of the side group (C**H**_3_O−) at 3.35 ppm, and (ii) for BuMA, the peak of the methylene group closest to the ester was used (CH_3_CH_2_CH_2_C**H**_2_O−), which appears at 3.9 ppm.

### Visual Tests

The visual changes of diluted and concentrated solutions in PBS were determined by observing the visual changes from 20 to 80 °C, every 1 °C, by using an IKA RCT basic stirrer hotplate, a continuously stirred water bath, and an IKA ETS-D5 temperature controller.

### Ultraviolet–Visible Spectroscopy

An Agilent Cary UV–Vis Compact Peltier UV–Vis spectrometer was used to determine the CPs, i.e., the temperature at which the transmittance was 50%, of 1% w/w solutions in DI water. For this, temperature ramp measurements were performed with a heating rate 1 °C min^–1^, and data were collected every 1 °C at 550 nm. The same equipment was used for determining the absorbance and, thus, concentration of SF in the in vitro drug release experiments.

### Rheology

The rheological properties of the solutions in PBS at a range of temperatures and concentrations were recorded using a TA Discovery HR-1 hybrid rheometer equipped with a 40 mm parallel steel plate (996921) and a Peltier temperature control unit. The changes were recorded at (i) a heating rate of 1 °C min^–1^, (ii) an angular frequency (ω) of 1 rad s^–1^, and (iii) a strain (γ) of 1%.

### Dynamic Light Scattering

The diluted aqueous solutions (1% w/w) of both OEGMA300_15_-*b*-BuMA_26_-*b*-DEGMA_13_ and Pluronic F127 were analyzed using a Zetasizer Nano ZSP from Malvern Instruments Ltd. (Malvern, UK). The polymer solutions were tested under the following solvents: (i) PBS and (ii) D_2_O/PBS. The polymer solutions were tested without any further processing, i.e., filtration, to ensure that a direct comparison between DLS and SANS can be made. The DLS experiments were performed at 25 °C, while in the case of PBS solutions, the samples were also tested at 10 °C. The solutions in PBS were also subjected to temperature ramp measurements. In all the cases, the scattered light was collected at a backscatter angle of 173°. Each sample was analyzed three times, and the results reported are the mean hydrodynamic diameters (*d*_H_) that correspond to the maximum of the peak by intensity and by number. The data were analyzed using Zetasizer software (version 7.11) from Malvern Polyanalytical.

### Differential Scanning Calorimetry

Solutions in PBS of OEGMA300_15_-*b*-BuMA_26_-*b*-DEGMA_13_ and Pluronic F127 (15% w/w) were prepared three times (three polymer solutions for each polymer). Each sample was analyzed using DSC three times (nine results per polymer). For the analysis, the samples were placed in T-zero style hermetic aluminum pans (purchased from Thermal Instruments Ltd., UK). The DSC was performed using the DSC Q2000 (TA Instruments, UK) at a heating rate of 5 °C/min between 2 and 60 °C under a nitrogen atmosphere. The obtained data were analyzed using Universal Analysis 2000 software (TA Instruments Version 4.5A).

### Rheological Small-Angle Neutron Scattering

The polymer solutions at 15% w/w D_2_O/PBS of both OEGMA300_15_-*b*-BuMA_26_-*b*-DEGMA_13_ and Pluronic F127 were investigated by Rheo-SANS. Specifically, the self-assembly of the samples was tested at a range of temperatures while simultaneously recording the changes in the shear moduli to confirm gelation. As the measurements were performed in the linear viscoelastic area as discussed in more detail in this section, small mechanical perturbations were applied, which would not disrupt the rest of the configuration in the system as it is also indicated by the isotropic patterns in Figure 4.

These measurements were performed at the time-of-flight SANS instrument ZOOM at the ISIS pulsed neutron source at the Rutherford Appleton Laboratory (Didcot, UK), using source-to-sample and sample-to-detector distances of 4 m and a wavelength range of 1.5–16.5 Å. For this experiment, an Anton Paar rheometer (Physica MCR501) equipped with a special Searle–Couette (rotating stator) measuring geometry and manufactured in grade V titanium was utilized. The height and diameter of the fixed outer cup are 81 and 50 mm, respectively, with the rotating inner stator being 62 mm × 48 mm. This geometry requires volumes of sample ranging from 9.5 to 47 mL. The temperature ramp measurements were performed at constant strain (γ = 1%) and constant angular frequency (ω = 1 rad s^–1^), which are within the linear viscoelastic area (see Figure S1). During this Rheo-SANS experiment, the beam was always traveling through the center of the geometry, and the experiment was performed at temperature steps with the temperature kept constant during SANS data accumulation (6 min and 7 s for each step). Data were reduced using MantidPlot.^42^ The resulted SANS curves of OEGMA300_15_-*b*-BuMA_26_-*b*-DEGMA_13_ were fitted using the SasView software (version 5.0)^43^ with an elliptical cylinder model with a hardsphere factor up to 43 °C, while the BroadPeak model was used to fit the data at higher temperatures. When using the elliptical cylinder model, in order to improve the fit in the high-*Q* region, polydispersity equal to 0.15 has been added to the length of the cylinder up to 34 °C, while the polydispersity of the radius minor has been fixed at 0.15 from 35 to 43 °C. The scattering length density (SLD) value of the solvent (D_2_O/PBS) was fixed at 6.36 × 10^–6^ Å^–2^, and the SLD of the polymer was fixed at 0.64 × 10^–6^ Å^–2^. The resulted SANS curves of Pluronic F127 were fitted using the small-angle diffraction tool in IRENA in IGOR software to fit the data. IGOR is a commercial program, and IRENA is a set of macros for it developed by Ilavsky and Jemian.^44^ All the peaks were fitted as Lorentzians.

### Cryogenic Scanning Electron Microscopy

The gel structure was imaged under cryogenic temperature using a Zeiss Auriga (Carl Zeiss, Germany) scanning electron microscope (cryo-SEM) equipped with a Quorum cryo-preparation chamber (PP3010T, Quorum Technologies, UK). 5 μL of polymer solution (15% w/w in PBS) was filled in a stacked and glued pair of brass rivets functioning as sample carrier, and gelation was promoted at 37 °C in an incubator. The samples were vitrified by plunging in slushed nitrogen (−200 °C) and transferring to the cryopreparation chamber for freeze fracture (−140 °C, by knocking off the top rivet with a cooled knife) to reveal the gels’ internal structure. Subsequently, free water was sublimated (−90 °C for 9 min), and the samples were finally sputter-coated with Pt (−150 °C, 5 mA, 30 s). After vacuum transfer to the microscope, images were recorded at −140 °C at a 2 kV acceleration voltage and 4 mm working distance using an in-lens detector. The porosity was determined as the inverse area fraction of binarized images (*n* = 3 per gel) using ImageJ software.

### Cryogenic Transmission Electron Microscopy

For cryo-TEM of the polymer solutions (15% w/w in PBS) at r.t., 5 μL of sample was applied to a glow-discharged Lacey carbon grid (EM Resolutions, UK) inside the chamber of a Leica GP2 plunge-freezing device held at 20 °C. Excess sample was blotted off in two 5 s blotting steps before plunging in liquefied propane/ethane mixture. Samples were imaged using a Gatan 626 cryoholder on a JEOL 2200FS TEM with a Gatan K2 direct electron detector. To image the samples at the gel state, they were manually blotted while being held outside the chamber and then incubated inside the chamber at 37 °C for 200 s before plunging into the propane/ethane mixture as for the r.t. samples.

### Preparation of Polymer Solutions Containing Sodium Fluorescein

The samples used during the in vitro drug release and the ex vivo intracameral injections were loaded with SF at 1 mg/mL. The polymer solution was prepared at 15% w/w in PBS. The homogeneous polymer solution was then mixed with SF at 1 mg/mL. The sink conditions, i.e., the concentration of the drug at maximum 20% (or 10% for perfect sink conditions) of the drug’s solubility,^45^ were respected as the solubility of SF in water is 500 mg/mL.

### In Vitro Drug Release

The solutions of the polymers (0.5 mL of 15% w/w in PBS), containing 1 mg/mL SF, were placed in 48-well plate and incubated at 37 °C to promote gelation. Once gelation was visually confirmed, 0.5 mL of PBS at 37 °C was added as supernatant, and they were kept at 37 °C throughout the experiment. Samples were withdrawn from the supernatant at specific time intervals, and they were analyzed via UV–Vis in order to determine the concentration of SF in the supernatant. The cumulative drug release (%) was calculated using eq 1 below. The drug loading content (%) and the encapsulation efficiency (%) were calculated using eqs 2(46) and 3 below.^46^
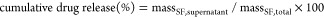
1
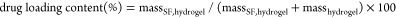
2
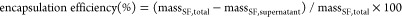
3

### Cell Toxicity Studies

ARPE-19 cells were grown at 37 °C and 5% CO_2_ in DMEM/F12 medium supplemented with 10% fetal calf serum, 100 U/mL penicillin, and 100 mg/mL streptomycin. The cytotoxicity of OEGMA300_15_-*b*-BuMA_26_-*b*-DEGMA_13_ and Pluronic F127 on ARPE-19 cells was assessed by MTT assay. Untreated cells were used as a positive control as a reference of 100% cell viability, while PEI, a cytotoxic polymer, was used as a negative control. Cells were seeded on 96-well plates at a density of 1 × 10^4^ cells per well. After 24 h, the cells were treated with polymer solutions at different concentrations (25, 50, 125, 250, and 500 μg/mL) for a further 24 h. MTT solution (10%) was added to each well and left to incubate for 4 h under standard culture conditions. MTT solution and media were carefully aspirated, and the formed crystals were solubilized with 100 μL of DMSO. Absorbance was read at 560 nm using an OPTImax microplate reader (Molecular Devices). Background absorbance was subtracted from readings to obtain final optical densities. Each biological experiment was repeated three times while keeping the samples at the same position to account for the effect of well position on the cell viability. Within each biological experiment, each concentration was tested three times. In total, the data were analyzed nine times per concentration, and the results presented are the average of nine repeats.

### Light Microscopy

ARPE-19 cells were grown in Ibidi chamber slides at a density of 1 × 10^4^ cell/chamber exposed for 24 h to OEGMA300_15_-*b*-BuMA_26_-*b*-DEGMA_13_, Pluronic F127, and PEI. After 24 h, cells were fixed using 4% paraformaldehyde and washed twice with PBS. Once washed, cells were permeabilized with 0.2% Tween-80 solution (in PBS) for 10 min followed by three PBS washes and staining with 50 μL of Phalloidin-647 per well (1:40 dilution). After 45 min, cells were washed twice with 0.2% Tween-80 solution, waiting 5 min between each wash. Cells were rinsed twice with PBS, treated for 10 min with 300 μL of Hoechst solution (1:2000) per well, and rinsed three times with PBS. Two drops of ProLong Diamond mounting media was added to each well. The samples were left to dry in the dark for at least 24 h before imaging. The staining protocol is a modified procedure previously reported by Ridolfo et al.^47^

Samples were imaged on a Nikon Eclipse Ti2 inverted microscope with an EMCCD Flash4.0 camera (Hamamatsu) and a Plan Apo λ 60×/1.4 NA oil objective combined with a 1.5× zoom, with no binning, to give a 2048 × 2048 pixel image with an *x*,*y* pixel size of 72 nm and a *z*-step of 200 nm. Hoechst and Phalloidin-647 were visualized with a LED light source pE-4000 (CoolLED), 365 nm excitation /447/60 nm emission, and 635 nm excitation/692/40 nm emission, respectively. Image stacks were deconvoluted with Huygens software (Scientific Volume Imaging, The Netherlands), and figures were prepared in Icy software.^48^ Images shown are maximum intensity projections.

### Ex Vivo Experiments with Intracameral Injections

Bovine eyes were delivered from a local abattoir within 3 h. Appropriate randomization in experiments with intracameral injections was achieved by labeling each eye with a number and placing another set of labels with the same figures in a nontransparent black bag. The randomization was done by means of pulling labels out of the bag randomly.

Polymer solutions (15% w/w) of OEGMA300_15_-*b*-BuMA_26_-*b*-DEGMA_13_, Pluronic F127 (positive control as it forms gels at 15% w/w at physiological temperature), and Pluronic F68 (negative control as it does not form gels at 15% w/w at physiological temperature) were prepared by dissolving the required amounts of each polymer in 1 mg/mL SF in PBS solution and stirring overnight at 4 °C to form clear solutions. The OEGMA300_15_-*b*-BuMA_26_-*b*-DEGMA_13_ solution in PBS containing 1 mg/mL SF was kept in an incubator at 3 °C below its gelation temperature for 20 min prior to the intracameral injections. The solution of Pluronic F127 in PBS containing 1 mg/mL SF was stored in the fridge at 4 °C for 20 min before the injection due to its high viscosity when kept at r.t. The solution of Pluronic F68 in PBS containing 1 mg/mL SF was kept in the fridge at 4 °C for 20 min prior to the injection.

All nine ex vivo bovine eyeballs were kept at 37 °C (human body temperature) for at least 40 min prior to injection in the anterior chamber (0.05 mL; 21-gauge needle, 1 mL syringe). Three runs for each polymer solution were performed within the incubator at 37 °C. All experiments were recorded for 3.5 min using iPhone XS and performed in triplicate, and data were analyzed using GraphPad Prism 8.0.2 software, where *p* < 0.05 was used as the statistical significance criterion. The significance of the calculated mean values ± standard deviations was assessed using one-way analysis of variance (ANOVA) followed by Bonferroni post hoc test, where *p* < 0.05 was selected as the statistical significance criterion.

Then, the extent of fluorophore spreading in the anterior chamber was evaluated using ImageJ software (version 1.50i) at different time points (0, 1, 1.5, 2, 2.5, and 3 min) starting from the moment of the complete pull of the needle out of the anterior chamber for each run of each polymer solution tested.

### Injectability Experiments

The injectabilities of the solutions of OEGMA300_15_-*b*-BuMA_26_-*b*-DEGMA_13_ and Pluronic F127 at 15% w/w in PBS were evaluated at r.t.. To accomplish this, a 2 mL gas-tight glass syringe (Cadence Science, Inc.) containing the solution, fitted with hypodermic needles, was placed vertically as shown schematically in Figure S3. The following needles (diameter × length, mm) were used: 23G 1″ (0.6 × 25, Fine-Ject), 24G 1″ (0.55 × 25, Fine-Ject), 25G 1″ (0.5 × 25, Fine-Ject), 26G 1″ (0.45 × 25, Fine-Ject), and 27G 3/4″ (0.4 × 20 Henke-Ject). Two different weights, 200 and 500 g, were placed on top of the needle, with a resulting force applied for the injection equal to 2.0 and 4.9 N, respectively. The time of injection of 0.5 mL of solution was measured, and the injection rate (mL/min) was calculated. Each experiment was repeated six times, and the standard error was calculated.

## Results and Discussion

### Design of the In-House Synthesized Polymer

**Figure 1:**
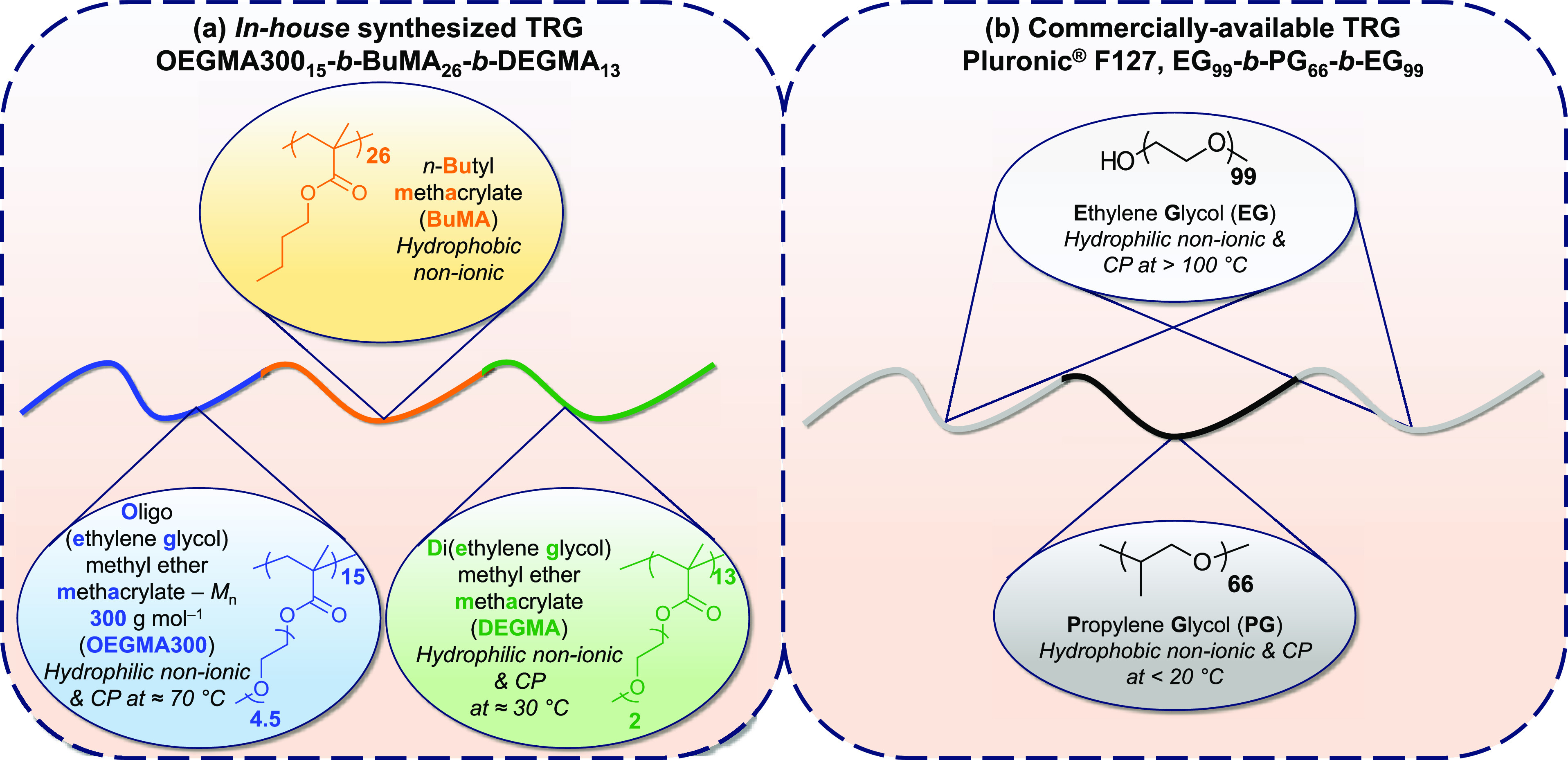
(a) Chemical structure of the in-house synthesized polymer, OEGMA300_15_-*b*-BuMA_26_-*b*-DEGMA_13_, and (b) chemical structure of the commercially available thermoresponsive polymer, Pluronic F127 or EG_99_-*b*-PG_66_-*b*-EG_99_.

To address the need for new thermoresponsive polymers, we designed and synthesized a triblock terpolymer that is based on a novel combination of methacrylate units.^100^ The triblock terpolymer has an ABC linear architecture and consists of two hydrophilic compartments based on OEGMA300 (A block, 40% w/w) and DEGMA (C block, also thermoresponsive close to physiological conditions: CP at 30 °C^40^, 25% w/w) and a hydrophobic central block based on BuMA (35% w/w) (Figure 1a). For comparison, the structure of Pluronic F127 is shown in Figure 1b. It is interesting to note that linear methacrylate polymers with PEG-based side chains did not show gelation^50,51^ as opposed to our novel combination of repeated units.^9^ We believe that the incorporation of the BuMA block is beneficial as it promotes self-assembly, while the OEGMA300 units balance the hydrophilicity of the structure and, thus, the solubility of the polymer in aqueous media. It is hypothesized that incorporating a DEGMA block, which responds close to physiological temperatures, and a hydrophilic OEGMA300 block might provide well-hydrated bridges between the polymer micelles, leading to hydrogel formation.

To ensure well-defined structural parameters, i.e., well-defined MM and composition, we have implemented the synthesis via GTP. These properties are crucial for controlling thermoresponsive properties, i.e., the CGC and the critical gelation temperature (CGT).^52^ The GPC and proton nuclear magnetic resonance (p^1^H NMR) analyses, as shown in Figures S2 and S3 and Table S1, reveal that we successfully synthesized the triblock terpolymer with a narrow MM distribution, as indicated by the low dispersity *Đ* of <1.15 and controllable MM and composition; its experimental structure is OEGMA300_15_-*b*-BuMA_26_-*b*-DEGMA_13_.

### Thermal Properties

**Figure 2:**
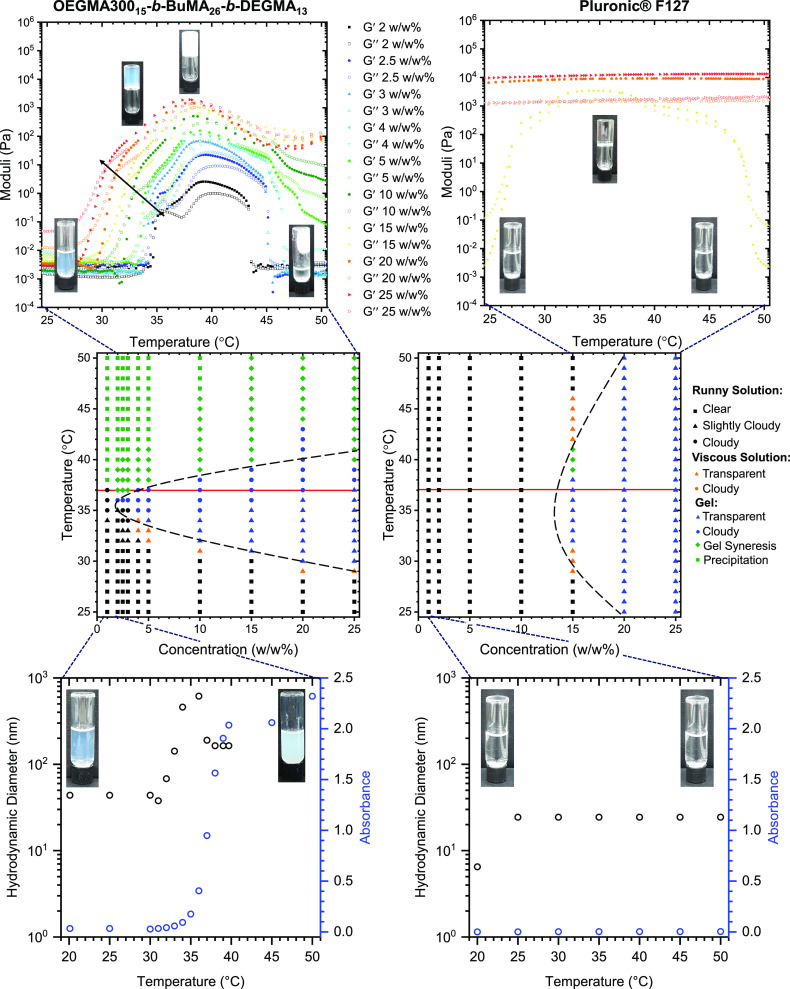
(Top) Variation of storage (*G*′, full symbol) and loss modulus (*G*″, empty symbol) as a function of temperature of the polymer solutions in phosphate-buffered saline (PBS) at various concentrations expressed in % w/w (OEGMA300_15_-*b*-BuMA_26_-*b*-DEGMA_13_, left; increased concentration is indicated by the black arrow and Pluronic F127, right; the data correspond to 15, 20, and 25% w/w). (Middle) Phase diagrams in PBS with the gelation approximately shown by a black dashed line (OEGMA300_15_-*b*-BuMA_26_-*b*-DEGMA_13_, left and Pluronic F127, right). (Bottom) Variation in hydrodynamic diameter (black circles) and in absorbance at 550 nm (blue circles) as a function of temperature for polymer solutions at 1% w/w in PBS (OEGMA300_15_-*b*-BuMA_26_-*b*-DEGMA_13_, left and Pluronic F127, right). Images of the visual inspection of the sol–gel transitions are also presented.

To investigate the macroscopic differences in aqueous media between OEGMA300_15_-*b*-BuMA_26_-*b*-DEGMA_13_ and Pluronic F127, we inspected the solutions in phosphate-buffered saline (PBS) across a range of temperatures and concentrations (Figure 2, top (rheology) and middle (visual test)). The gelation temperature (*T*_gel_), defined as the temperature at which (i) the sample does not flow upon tube inversion by visual tests and (ii) the storage modulus exceeds the loss modulus (*G*′ > *G*″) by rheology,^53^ is of main importance.

While all the samples of OEGMA300_15_-*b*-BuMA_26_-*b*-DEGMA_13_ are runny solutions at r.t. at the concentrations tested (1–25% w/w), the ones of Pluronic F127 are gels at r.t. when the concentration increases from 15 to 20% w/w as extensively reported in the literature.^54−57^ This is also confirmed by rheology as the loss modulus exceeds the loss modulus (*G*″ > *G*′) at r.t. for the samples of OEGMA300_15_-*b*-BuMA_26_-*b*-DEGMA_13_. On the other hand, the solution of 15% w/w Pluronic F127 is highly viscous at r.t. (*G*″ > *G*′) as indicated by the higher values in moduli, while it forms an elastic gel with increase of the concentration to 25% w/w (*G*′ > *G*″). The higher viscosity of the Pluronic F127 solution at 15% w/w compared to the one of OEGMA300_15_-*b*-BuMA_26_-*b*-DEGMA_13_ can be translated to the lower injectability rate of the former (Figure S4).

Gelation is promoted when the OEGMA300_15_-*b*-BuMA_26_-*b*-DEGMA_13_ solutions are heated close to b.t., with *T*_gel_ controllably decreasing from 36 to 30 °C as the concentration increases from 2 to 25% w/w; photographs of the solution state at r.t. and the gel state at b.t. are shown in Figure S5. Interestingly, the strength of the gels formed by OEGMA300_15_-*b*-BuMA_26_-*b*-DEGMA_13_ is strongly controlled by the polymer concentration as it increases by three orders of magnitude when the concentration is increased from 2 to 25% w/w (Figure S6) as opposed to the solutions of Pluronic F127, whose gels’ strength varies by one order of magnitude. Specifically, the maximum storage modulus is tuned from 3 to 1922 Pa when the concentration increases from 2 to 25% w/w, corresponding to Young’s modulus (*E*) values from 8 to 5765 Pa (*E* = 2*G*(1 + ν), with Poisson’s ratio (ν) = 0.5 for soft materials such as gels),^24,58,59^ which are comparable to previously reported ABC injectable hydrogelators with storage moduli starting from 25 Pa.^24^ This indicates that the OEGMA300_15_-*b*-BuMA_26_-*b*-DEGMA_13_ gels could serve as injectable systems starting from at least 2.5% w/w (maximum storage modulus at 22 Pa). The effect of concentration on the gel strength is attributed to higher physical cross-linking density with increased concentration, leading to increased stiffness as previously reported.^24^ The finely tunable stiffness of OEGMA300_15_-*b*-BuMA_26_-*b*-DEGMA_13_ is advantageous in tissue engineering applications as it is well reported that the stiffness of the material governs stem cell differentiation.^60−62^ On the other hand, the gels formed by Pluronic F127 are stronger (*G′* varying from 3500 to ∼13,000 Pa, concentration-dependent), and might be more suitable for 3D-printing applications, in which mechanically strong 3D-printed structures are required.

When the solutions of OEGMA300_15_-*b*-BuMA_26_-*b*-DEGMA_13_ are heated beyond their *T*_gel_, gel syneresis, i.e., slight exclusion of solvent from the gel,^63,64^ is detected followed by precipitation. Generally, the higher the concentration, the more stable the gel is in terms of the upper gelation boundary, indicating a concentration-dependent stability, which is also associated with longer stability at physiological temperature.

In addition to the previous findings regarding gelation, the diluted solutions of OEGMA300_15_-*b*-BuMA_26_-*b*-DEGMA_13_ (1% w/w in PBS) present a CP at 37 °C, caused by the aggregation of the polymeric micelles (Figure 2, bottom left), while the one of Pluronic F127 shows no visual thermoresponse (Figure 2, bottom right).

Importantly, we report here a polymer that (i) gels at concentrations seven times lower than Pluronic F127, (ii) exhibits low viscosity at r.t., which enables the use of narrow needles, minimizing trauma and benefitting patient safety, and (iii) shows controllable stiffness, which is advantageous in tissue engineering applications in which stem cell differentiation is desired.

**Figure 3:**
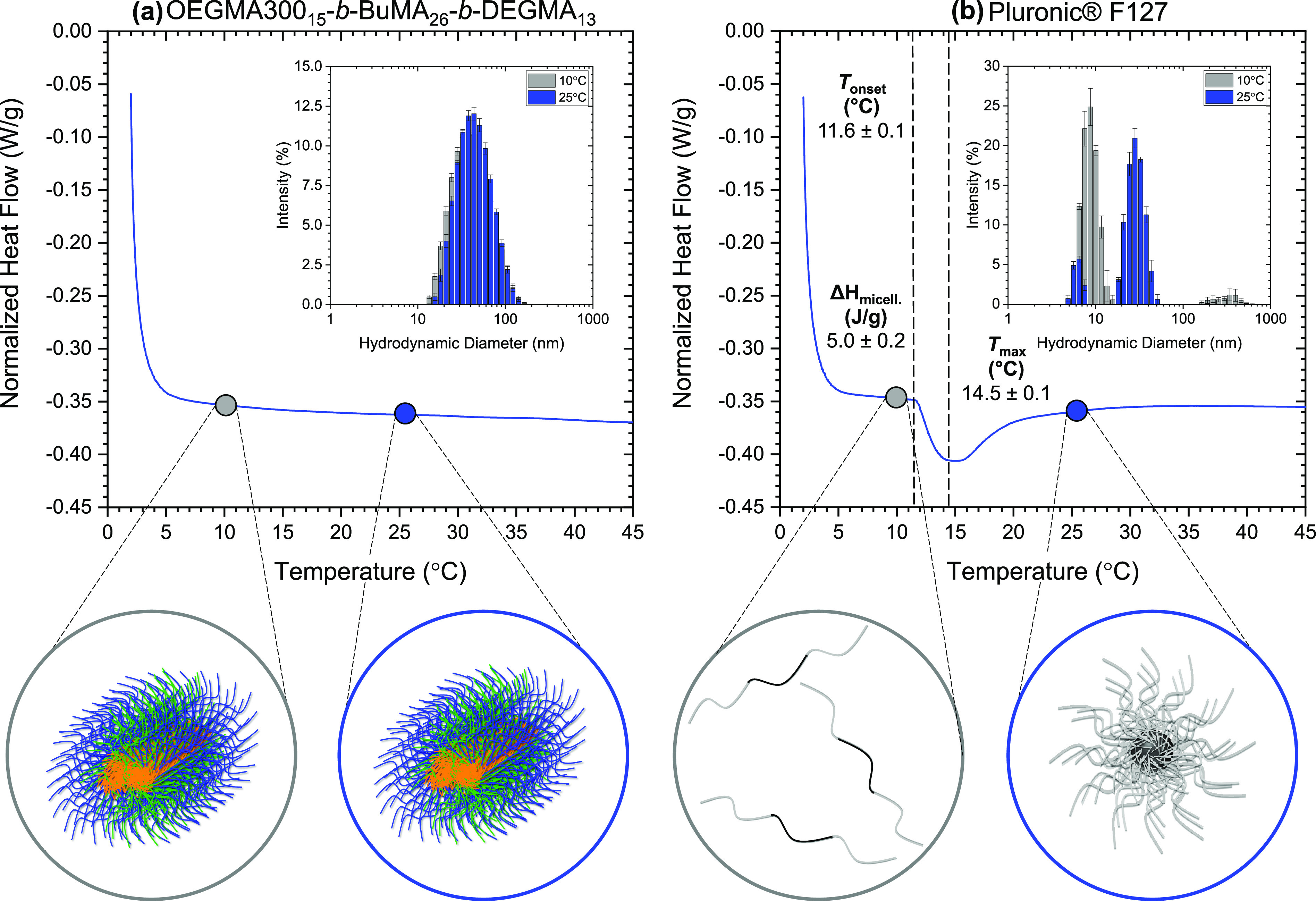
DSC thermograms of 15% w/w polymer solutions in phosphate-buffered saline (PBS) of (a) OEGMA300_15_-*b*-BuMA_26_-*b*-DEGMA_13_ and (b) Pluronic F127. The DLS histograms of 1% w/w polymer solutions in PBS at 10 °C (gray) and 25 °C (blue) are also presented as insets. Inserts show the structures at 10 °C (gray circle) and 25 °C (blue circle), in which the temperature-driven self-assembly of Pluronic F127 is indicated.

In order to further investigate the thermal behavior of the polymer solutions, we employed DSC, which is a well-documented technique to monitor the micellization, gelation, and CP of polymer solutions.^57,65−70^ We chose a polymer concentration of 15% w/w, which corresponds to the GCG of Pluronic F127. The DSC thermogram of a 15% w/w OEGMA300_15_-*b*-BuMA_26_-*b*-DEGMA_13_ solution in PBS (Figure 3a and Figure S7) shows no apparent changes in the heat flow up to 45 °C. This is attributed to the amphiphilic nature of the polymer, which promotes self-assembly in aqueous solutions: a central BuMA-based hydrophobic block and two outer hydrophilic blocks (OEGMA300- and DEGMA-based). In contrast, a broad endothermic peak is present on the DSC thermogram of a 15% w/w Pluronic F127 solution (Figure 3b) with an onset temperature (*T*_onset_) at 11.6 ± 0.1 °C. This peak is indicative of the micellization process of Pluronic F127 caused by the dehydration of the PG units, and it has been previously reported in the literature.^65,68^ The enthalpy of micellization (Δ*H*_micell_) is 5.0 ± 0.2 J/g (equal to 63 kJ/mol). Both the temperature of the maximum of the peak (*T*_max_) and the Δ*H*_micell_ are in good agreement with previously reported values for similar systems.^57,65−68^ As previously stated, the gelation process is scarcely endothermic (i.e., almost athermal), and thus, it has only been observed as a spike on the main peak at concentrations higher than the ones in the present study.^65,68,69^ Studies on different systems other than Pluronics recorded the phase separation at higher concentrations via DSC.^70^

To confirm the hypothesis of micellization occurring at ∼12 °C, we carried out DLS analysis on both polymers at 1% w/w in PBS at 10 and at 25 °C (insets in Figure 3 and Table S2). Concerning OEGMA300_15_-*b*-BuMA_26_-*b*-DEGMA_13_, we observed micelles (*d*_H_ ≈ 40 nm) at both temperatures, while for Pluronic F127, we observed a transition from unimers (*d*_H_ ≈ 9 nm) to micelles (*d*_H_ ≈ 30 nm) when the temperature is increased from 10 to 25 °C, which is in good agreement with the literature.^67,71,72^ These results were corroborated by measuring the absorbance of the polymer solution at 550 nm (insets in Figure 3).

### Gelation Mechanism

OEGMA300_15_-*b*-BuMA_26_-*b*-DEGMA_13_ and Pluronic F127 exhibit clear differences in their macroscopic gelation properties as well as in their thermal and self-assembly behavior, which prompted us to use SANS to gain further insights into their nanoscale self-assembly behavior. While recording the neutron scattering profiles of the polymeric ensembles at the nanoscale across a temperature range, we simultaneously measured their macroscopic rheological properties to confirm gelation. In this study, we report and discuss the SANS analysis of the polymer solutions at 15% w/w as this is the CGC of Pluronic F127. The solutions were prepared in deuterated phosphate-buffered saline (D_2_O/PBS) to achieve good neutron contrast; the gelation area is slightly wider than in PBS (Figures S8 and S9).

**Figure 4:**
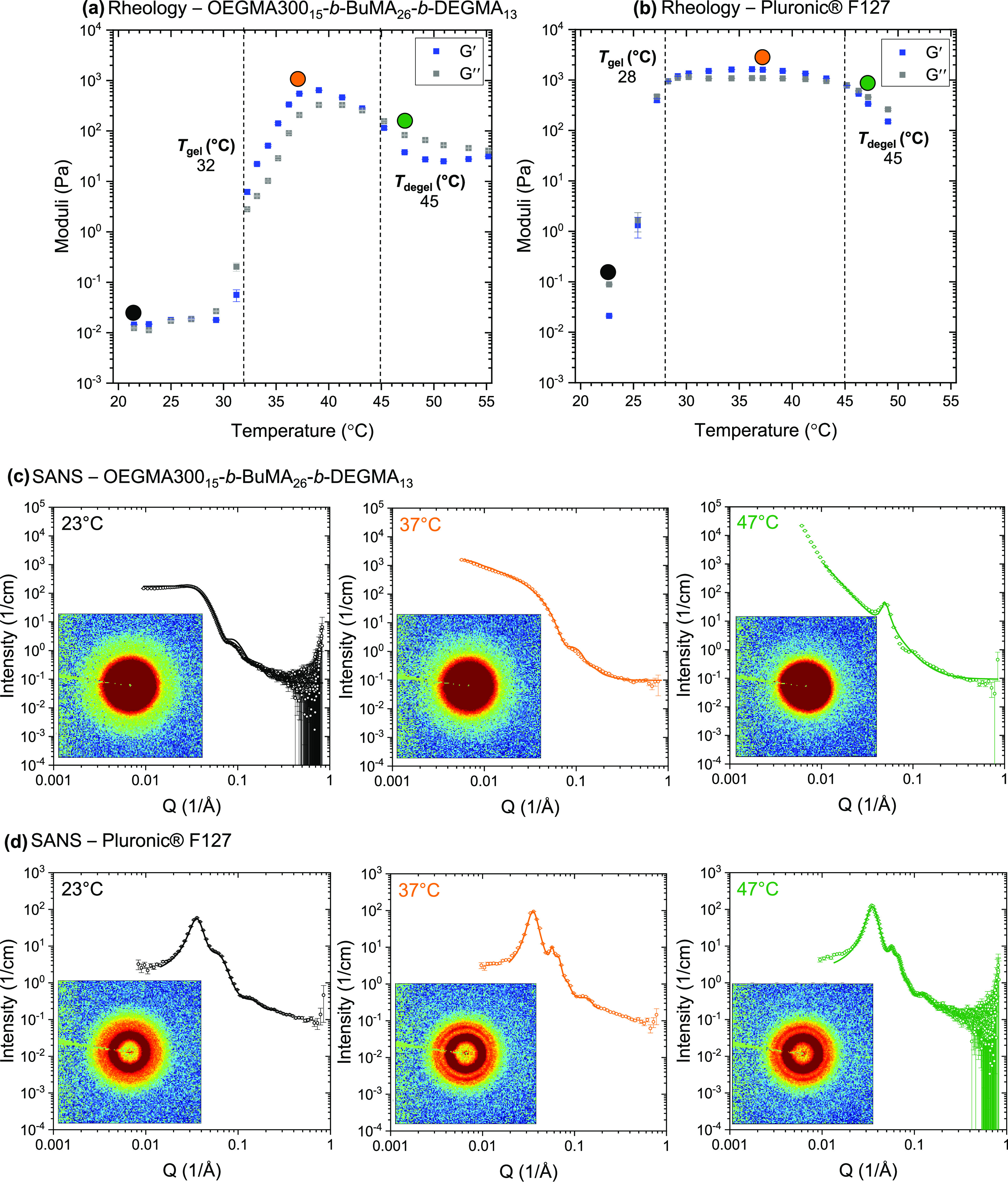
(Top) Temperature sweep rheological measurements on (a) OEGMA300_15_-*b*-BuMA_26_-*b*-DEGMA_13_ and (b) Pluronic F127 (storage modulus, *G*′, in blue and loss modulus, *G*″, in gray).(Middle) SANS data from OEGMA300_15_-*b*-BuMA_26_-*b*-DEGMA_13_ (c) at selected temperatures, which were fitted by using SasView software, an elliptical cylinder model with a hard sphere at 23 and 37 °C, and a BroadPeak model at 47 °C. (Bottom) SANS data from Pluronic F127 (d), which were fitted by using the small-angle diffraction tool in IRENA in IGOR software. The corresponding 2D data are also presented. The experiments were performed on 15% w/w solutions in deuterated phosphate-buffered saline (D_2_O/PBS).

As corroborated by rheology, Figure 4, both samples are in the liquid phase at r.t., while they form gels as the temperature increases to the critical *T*_gel_ corresponding to *G*′ > *G*″.^53^ Interestingly, both samples are in the gel state at b.t. (*T*_gel, OEGMA30015-*b*-BuMA26-*b*-DEGMA13_ = 32 °C, *T*_gel,Pluronic F127_ = 28 °C), while the gels are destabilized (*G*″ > *G*′, *T*_degel_) at ≥45 °C as expected from the visual tests (Figure 2, middle).

The in-house synthesized polymer, OEGMA300_15_-*b*-BuMA_26_-*b*-DEGMA_13_, presents different SANS profiles as the temperature increases (Figure 4c and Figures S10, S11, S12, and S15a), indicating changes in the morphology of its self-assembled structures. We used an elliptical cylinder model to fit the data up to 43 °C. To account for the interparticle interference in concentrated solutions, the hard-sphere structure factor, i.e., repulsive short-range interaction with excluded volume,^73^ was used. A BroadPeak model was used to fit the data from 45 °C; this temperature coincides with the *T*_degel_ by rheology.

**Figure 5:**
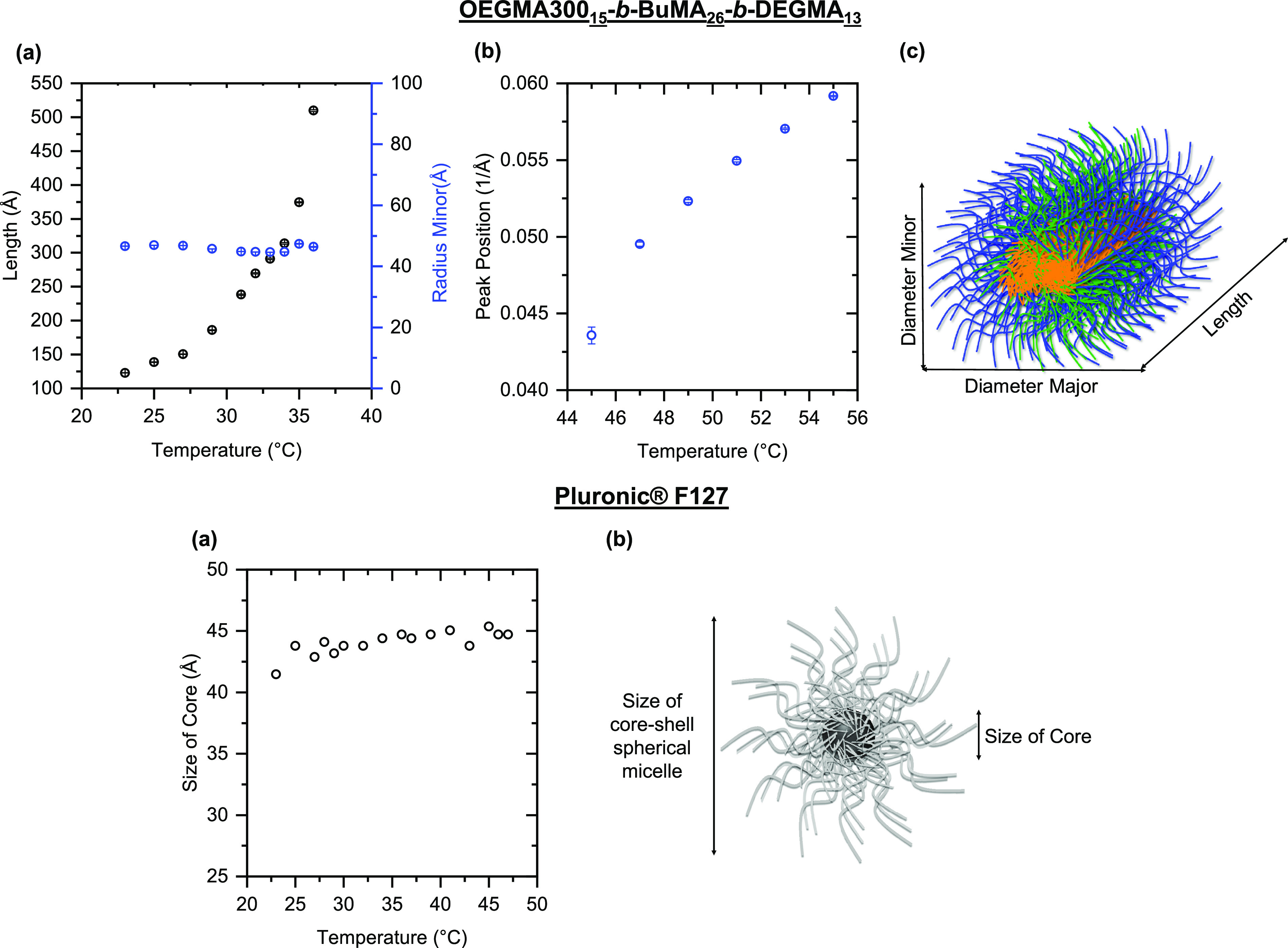
The fitting parameters of the self-assembled structures as a function of temperature for OEGMA300_15_-*b*-BuMA_26_-*b*-DEGMA_13_ (top panel): (a) length or radius minor vs temperature, (b) peak position *vs* temperature, and (iii) micelle structure adopted by OEGMA300_15_-*b*-BuMA_26_-*b*-DEGMA_13_, in which blue, orange, and green represent the hydrophilic, hydrophobic, and thermoresponsive blocks, respectively; Pluronic F127 (bottom panel): (a) size of core vs temperature and (b) globular structure adopted by Pluronic F127, where gray and black correspond to the hydrophilic and hydrophobic blocks, respectively.

From the SANS data, we inferred that the OEGMA300_15_-*b*-BuMA_26_-*b*-DEGMA_13_ formed micelles shaped as elliptical cylinders, whose best-fit radius minor is around 45 to 47 Å (blue dots in Figure 5a, top panel), while the best-fit axis ratio is around 1.3 to 1.5 (Figure S16a), thus leading to a best-fit radius major of around 55 to 65 Å (Figure S16b) at a temperature range from 23 to 36 °C. Interestingly, we observed a clear trend for the length of the cylinder, which increases significantly (from 123 to 510 Å) within the same temperature range (black dots in Figure 5a, top panel); the fitting parameters of the elliptical cylinder above 36 °C are not presented in Figure 5 as the best-fit length was outside the limits of the SANS technique (2000 Å).

In order to fit the SANS data for OEGMA300_15_-*b*-BuMA_26_-*b*-DEGMA_13_ above the *T*_degel_, we used a BroadPeak model, which provided the best-fit position of the Bragg peak.^74^ This model is a combination of a Lorentzian peak function and a power law decay and could suggest the presence of a bicontinuous structure^75^ above the *T*_degel_. As observed, the obtained Bragg peak position shifted from 0.044 Å^–1^ at 45 °C to 0.059 Å^–1^ at 55 °C (Figure 5b, top panel). The *d*-spacing of this peak, calculated as *d* = 2π/*Q*, is a characteristic distance between the scattering inhomogeneities,^75^ and it decreases from *d* = 144.21 Å and *d* = 106.19 Å as the gel syneresis progresses from 45 to 55 °C.

A schematic of the elliptical cylinder structure adopted by OEGMA300_15_-*b*-BuMA_26_-*b*-DEGMA_13_ is also proposed (Figure 5c, top panel), in which the hydrophilic OEGMA300 blocks and the hydrophilic and thermoresponsive DEGMA blocks, shown in blue and green, respectively, extend from the hydrophobic BuMA core, shown in orange, toward the aqueous environment.

The SANS patterns from Pluronic F127, (Figure 4d and Figures S13, S14, and S15b) are in a good agreement with the ones previously reported in the literature for concentrated solutions of the same polymer.^57,76−80^ We used the small-angle diffraction tool in IRENA in IGOR software to fit the scattering as it was found to capture the scattering features/peaks best.

Interestingly, the scattering patterns from the solution of Pluronic F127 present a series of strong structural peaks/shoulders in the low-*Q* region. These are characteristic of a concentrated system of spherical aggregates, as previously reported in both Pluronic^80^ and Tetronic (X-shaped polymers with four-arm PG_*x*_-*b*-EG_*y*_)^81^ systems. The first peak at *Q* ≈ 0.035 Å^–1^ is well-distinguishable at all temperatures, (Figure 4d), and it is due to interparticle interference.^77^ We observed a shoulder at *Q* ≈ 0.061 Å^–1^ below 25 °C, at which the sample is a free-flowing liquid. At higher temperatures, a clear peak is detected at *Q* ≈ 0.057 Å^–1^ (Figure 4d, middle and right), followed by two shoulders at *Q* = 0.067 Å^–1^ and *Q* = 0.085/0.094 Å^–1^ at higher temperatures. The sharpening of the peaks observed at higher temperatures reveals that highly structured nanoparticles with long-range order are present, as previously observed.^80^ These features are related to the interparticle interference and are associated with the formation of a macrocrystalline network.^57,77,78,80^

The mechanism of the macrocrystalline network formation reported in the literature is controversial, with studies reporting simple cubic,^78^ body-centered-cubic (BCC)^66,79^ and face-centered-cubic (FCC)^57,80^ lattice structures. The ratio of the peaks to the first-order peak (*Q*/*Q*_0_) is an indication of the lattice structure. For example, a ratio of  corresponds to a BCC structure,^82^ while a ratio of  corresponds to an FCC structure.^83^ In the current study, when the data are examined carefully, it can be seen that the second-order peak (*Q* ≈ 0.0385 Å^–1^) is smeared with the first-order peak as previously reported by Prud’homme et al.^78^ and Li et al.^77^ Taking this into consideration as well as the peak positions listed in Table S3, it is suggested that an FCC structure is present in the current system. However, one should bear in mind that detailed examination and consideration of other parameters, such as the volume fraction,^77,78^ should be taken into account for determining the lattice structure.

In addition to the series of peaks/shoulders at low *Q* values, a broad shoulder arising from a spherical form factor is present at *Q* ≈ 0.12 Å^–1^. It is well documented that Pluronic F127 forms core-shell spherical micelles with well-hydrated PEG coronas and dehydrated PPG cores.^80,84−86^ This smaller feature at high *Q* is due to intraparticle interference^77^ and corresponds to the size of the core of the micelles. We obtained a best-fit size core for the micellar core of approximately 4.4 nm, similarly to previously reported values.^77,80^ This shoulder remains unchanged over the temperature range tested, and it is an indication that the size of the self-assembled structures of Pluronic F127 is not affected by the temperature as previously reported.^78^ This can also be seen in Figure 5a (bottom panel), which presents the independence of the size of the core as a function of temperature. The suggested core-shell spherical structure is shown in Figure 5b (bottom panel), in which the well-hydrated PEG corona is shown in gray and the compact hydrophobic core is illustrated as a black sphere.

**Figure 6:**
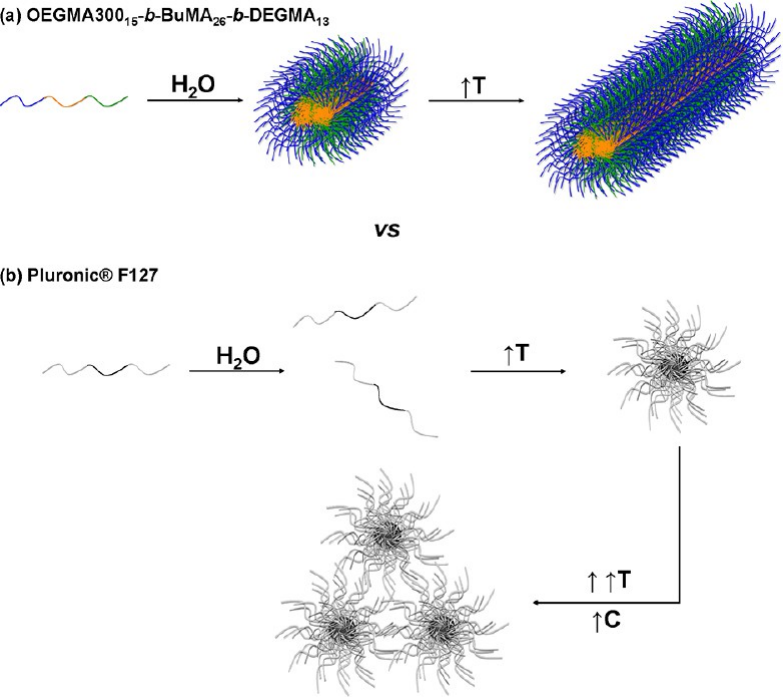
Schematic illustration of the proposed mechanisms of gelation for (a) OEGMA300_15_-*b*-BuMA_26_-*b*-DEGMA_13_ (top) and (b) Pluronic F127. The blocks of OEGMA300, BuMA, DEGMA, EG, and PG are colored in blue, orange, green, gray, and black, respectively. The macrocrystalline lattice structure adopted by Pluronic F127 is not shown for simplicity.

Our extensive SANS analysis allowed us to reveal the nanoscale differences between OEGMA300_15_-*b*-BuMA_26_-*b*-DEGMA_13_ and Pluronic F127. The formation of gel by OEGMA300_15_-*b*-BuMA_26_-*b*-DEGMA_13_ is caused by the growth of micelles to cylindrical/wormlike micelles, as indicated by the significant increase in their length (Figure 5). On the other hand, the temperature-driven micellization of Pluronic F127 is caused by the thermoresponse of PG units, as revealed by DSC and DLS analyses, while its gelation is driven by the close packing of the micelles into a macrocrystalline network. The proposed gelation mechanisms are shown schematically in Figure 6, where the tricomponent system (OEGMA300_15_-*b*-BuMA_26_-*b*-DEGMA_13_) is shown in blue, orange, and green (top), while Pluronic F127, which is a bicomponent system (EG_66_-*b*-PG_99_-*b*-EG_66_), is presented in gray and black (bottom).

### Gel Structure

**Figure 7:**
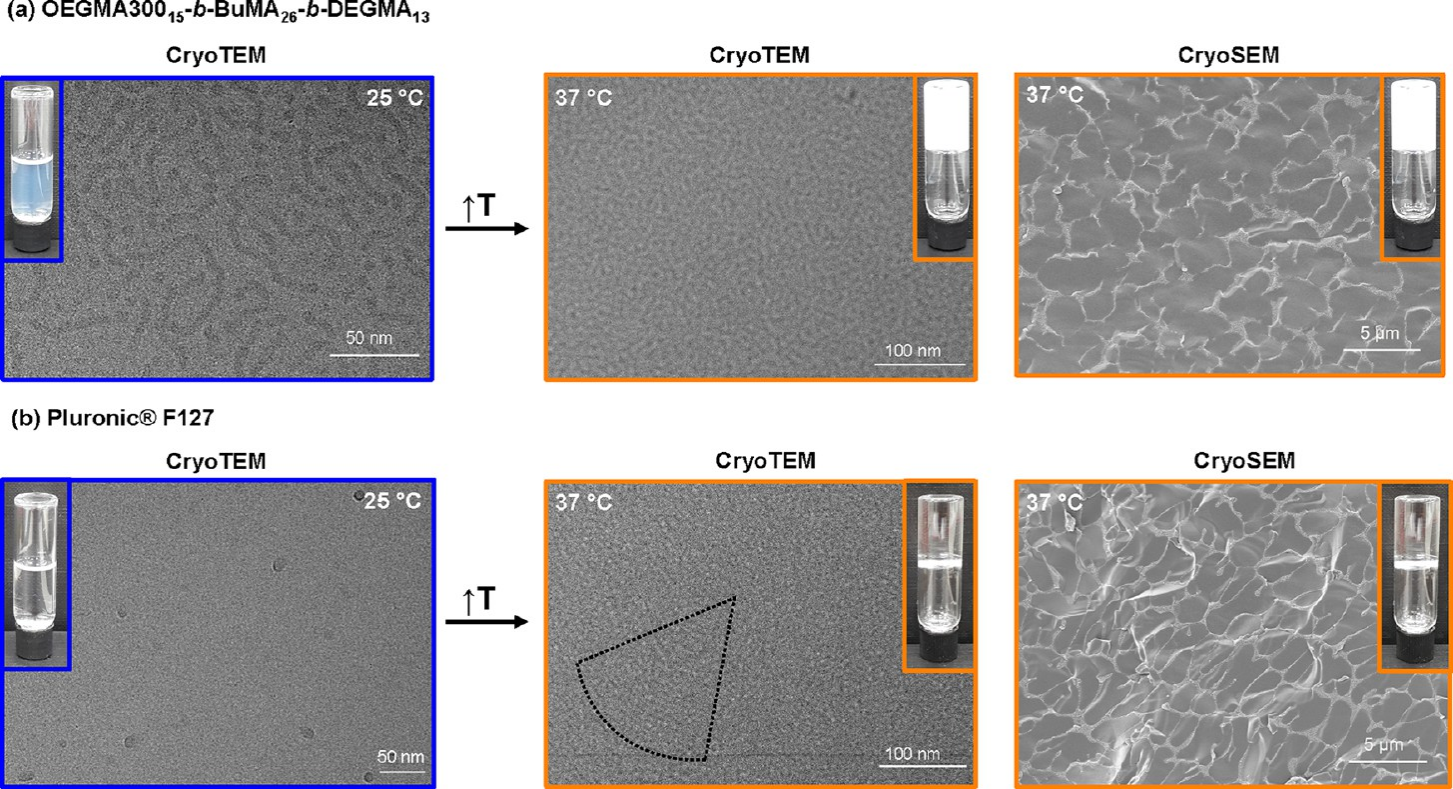
Electron microscopy images of the samples at 15% w/w in phosphate-buffered saline (PBS) at the solution state (25 °C, left, blue) and at the gel state (37 °C, middle and left, orange): (a) OEGMA300_15_-*b*-BuMA_26_-*b*-DEGMA_13_ and (b) Pluronic F127 (the dotted line on the CryoTEM image of Pluronic F127 at 37 °C is to guide the eye to the alignment of the micelles into rows). Insets show photographs of inverted vials. Note that the solution flows when the vial is inverted, while when a gel is formed, no flow is observed.

The liquid samples at 15% w/w in PBS at r.t. were imaged via cryo-TEM, while the gel structure formed at 37 °C was visualized via both cryo-TEM and cryo-SEM (Figure 7). Individual tubular structures (worm-like structures) with diameters of around 8 nm are observed at r.t. for OEGMA300_15_-*b*-BuMA_26_-*b*-DEGMA_13_, which is in good agreement with the Rheo-SANS data. Dense small spherical micelles measuring around 14 nm are observed for Pluronic F127. When heated up to the gel state, Pluronic F127 appears from both cryo-TEM and cryo-SEM to form a dense and well-ordered porous structure, as previously reported,^87^ while OEGMA300_15_-*b*-BuMA_26_-*b*-DEGMA_13_ is less organized and could be characterized as amorphous. A microporous, interconnected 3D network structure was confirmed via cryo-SEM for both gels, with the size of pores and width of polymer scaffold found widely homogeneous and comparable between both materials at the lower micrometer size regime. While the pores in the gel of Pluronic F127 are elongated, the ones formed in the OEGMA300_15_-*b*-BuMA_26_-*b*-DEGMA_13_ gel are more spherical. In addition, higher porosity is observed for OEGMA300_15_-*b*-BuMA_26_-*b*-DEGMA_13_ (80.6 ± 0.5%) compared to the one of Pluronic F127 (72.2 ± 0.5%), which is essential for biomedical applications involving drug encapsulation and release and those that require efficient oxygen and nutrient transport through offering a high level of free void space. It is noteworthy that in the gel formed by Pluronic F127, the micelles are being organized, resulting in rows of micelles (Figure 7, bottom middle, and Figure S17) as previously observed.^88^

### In Vitro and ex Vivo Applications

The in vitro gelation of polymer solutions at 15% w/w in PBS containing SF at 1 mg/mL was investigated to explore the potential of OEGMA300_15_-*b*-BuMA_26_-*b*-DEGMA_13_ hydrogels for drug delivery. Both solutions of OEGMA300_15_-*b*-BuMA_26_-*b*-DEGMA_13_ and Pluronic F127 gelled instantly upon injection to PBS at 37 °C (Figure S18).

**Figure 8:**
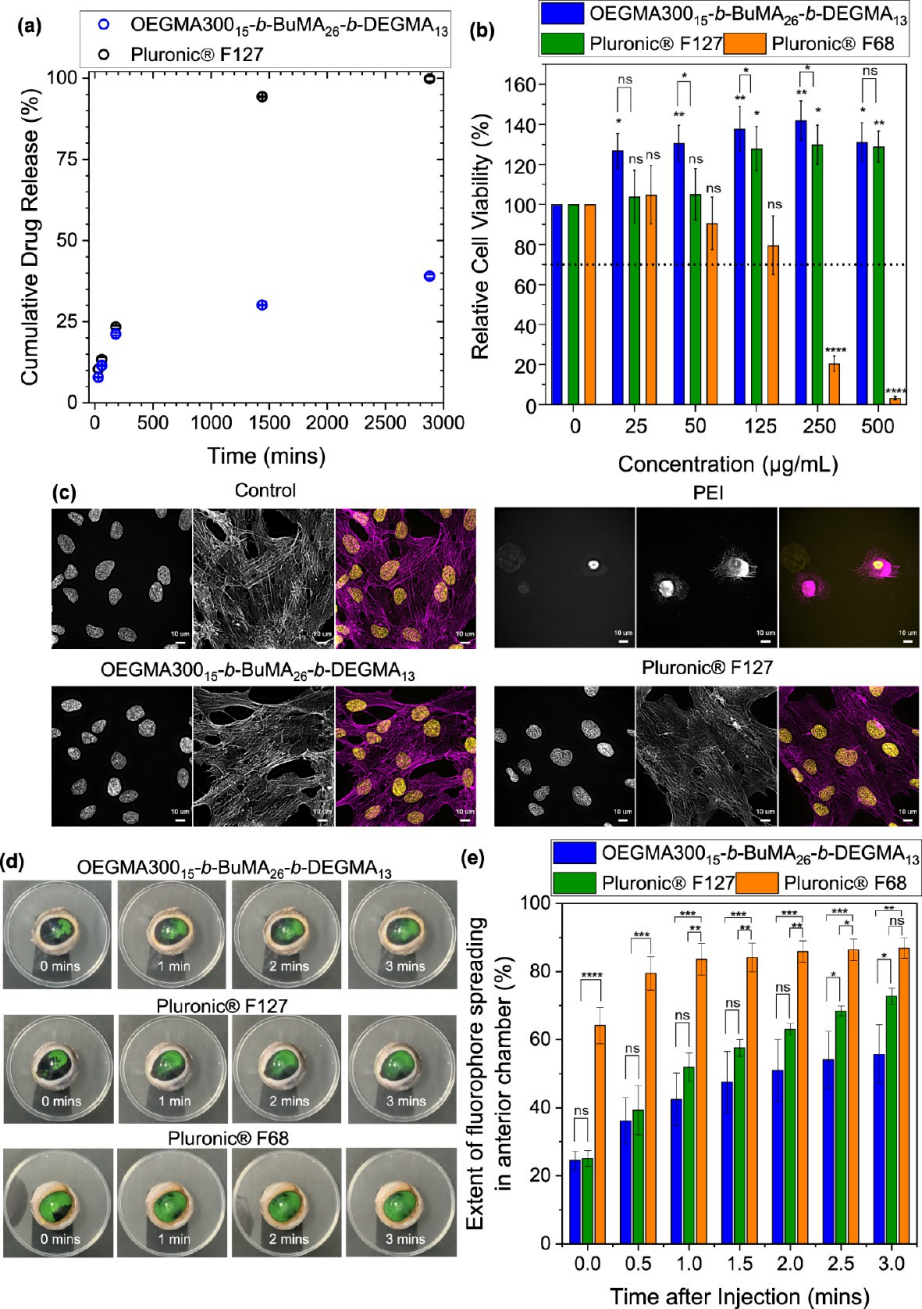
(a) In vitro sodium fluorescein (SF) release profile from 15% w/w gels in phosphate-buffered saline (PBS). (b) Relative ARPE-19 cell viability as a function of polymer concentration. (c) Light microscopy images of ARPE-19 cells when untreated (positive control) or exposed for 24 h to 125 μg/mL of the polymer solutions; nuclei (left) and cytoplasm (center, F-actin staining) are in grayscale, and the resulting overlay is in color (right; scale bars = 10 μm). (d) Ex vivo intracameral injections of 15% w/w solutions in PBS containing 1 mg/mL SF into bovine eyes. (e) Extent of SF spreading in anterior chamber as a function of time.

We observed sustained short-term drug release from both gels, with OEGMA300_15_-*b*-BuMA_26_-*b*-DEGMA_13_ releasing the model drug at a slightly lower rate compared to Pluronic F127 (Figure 8a). While long-term controlled drug release is observed for OEGMA300_15_-*b*-BuMA_26_-*b*-DEGMA_13_, a burst release is detected for Pluronic F127 at 24 h due to gel destabilization, as previously observed and reported for this system.^89−91^ Interestingly, higher drug loading content and encapsulation efficiency are detected for OEGMA300_15_-*b*-BuMA_26_-*b*-DEGMA_13_ (Figure S19), confirming this system’s superior properties in the controlled drug release field.

Both polymers showed good cell compatibility when tested in ARPE-19 cells (Figure 8b). Importantly, OEGMA300_15_-*b*-BuMA_26_-*b*-DEGMA_13_ shows comparable biocompatibility with Pluronic F127 at the lowest concentration tested (25 μg/mL), while it outperforms Pluronic F127 at high concentrations. It should be noted that cell viability is calculated relative to the control, which was kept constant at 100%; thus, some viability values are surpassing the value of 100% as previously observed.^92^ This is attributed to the experimental setup, i.e., treatments were performed in the same well positions during repeats for consistency as the cell growth in 96-well plates depends on the well position.^92^ PEI was used as the negative control as it is known to be toxic.^93,94^ As expected, PEI exposure shows toxic effects at concentrations of 125 μg/mL, which significantly increase with concentration. These findings were also confirmed by fluorescence staining and light microscopy (Figure 8c), in which the results are compared to the ones of untreated cells as a reference of 100% cell viability (positive control). Extensive cellular proliferation was observed when cultured in the presence of OEGMA300_15_-*b*-BuMA_26_-*b*-DEGMA_13_ and Pluronic F127, confirming their biocompatible nature. In contrast, low cell number and a lack of cellular organization are observed in the case of PEI.

To evaluate the applicability of OEGMA300_15_-*b*-BuMA_26_-*b*-DEGMA_13_ for drug delivery applications, 15% w/w solutions containing 1 mg/mL SF were injected in ex vivo bovine eyes, and the fluorophore distribution was analyzed over time. In these experiments, the 15% w/w solution of Pluronic F127 was used as a positive control since it can form gel at physiological temperature, and the 15% w/w of Pluronic F68 was used as a negative control (does not form a gel at physiological temperature). Both control solutions also contained 1 mg/mL SF. All solutions were injected intracamerally into the anterior chamber of freshly excised bovine eyes thermostated at physiological temperature, and the spreading of SF was monitored visually using video recording (see sample videos in the Supporting Information) and analyzed using image analysis (Figure 8d,e and Figure S20). As expected, the negative control formulation based on Pluronic F68 exhibited a rapid SF release in the anterior chamber due to the absence of gelation. Both OEGMA300_15_-*b*-BuMA_26_-*b*-DEGMA_13_ and Pluronic F127 formulations exhibited significantly slower SF release (*p* < 0.05) compared to Pluronic F68, which is related to their gelation in the anterior chamber. It is interesting to note that the formulation based on OEGMA300_15_-*b*-BuMA_26_-*b*-DEGMA_13_ shows a significantly slower release rate of SF compared to Pluronic F127 (*p* < 0.05).

Taking the ex vivo drug release results into consideration, both polymers are proven to be suitable candidates for future in vivo applications in controlled ocular drug delivery. Despite its nondegradable nature, due to the absence of hydrolyzable or biodegradable bonds on the polymer backbone, Pluronic F127 is currently the only thermogel applied in clinical trials.^1^ Thus, biodegradability does not seem to be a necessity for clinical applications. We hypothesize that OEGMA300_15_-*b*-BuMA_26_-*b*-DEGMA_13_, despite its nondegradable nature, will be dissolved in the plasma and excreted in urine as its MM (∼10 kDa) is lower than the kidney’s molecular weight cutoff (MWCO_kidneys_ ≈ 30–50 kDa),^95^ with its glomerular filtration rate being dependent on its MM.^96^ As such, OEGMA300_15_-*b*-BuMA_26_-*b*-DEGMA_13_ is suitable for the design of injectable delivery systems to the anterior chamber and, specifically, in applications where longer drug residence will be of great importance. This potentially indicates a further advantage of this polymer compared to Pluronic F127.

## Conclusions

In conclusion, we report here a new thermoresponsive, biocompatible terpolymer, OEGMA300_15_-*b*-BuMA_26_-*b*-DEGMA_13_, that gels under physiological conditions at a concentration that is seven times lower than the one needed for Pluronic F127. While OEGMA300_15_-*b*-BuMA_26_-*b*-DEGMA_13_ inherently forms micelles due to the incorporation of a permanently hydrophobic block, the micellization of Pluronic F127 is temperature-dependent and driven by the thermoresponse of the PG units. We used state-of-the-art characterization techniques such as DSC and SANS to probe the self-assembled nanostructures and gain insights into the gelation mechanism of OEGMA300_15_-*b*-BuMA_26_-*b*-DEGMA_13_. Based on our findings, we conclude that OEGMA300_15_-*b*-BuMA_26_-*b*-DEGMA_13_ forms a gel due to the growth of the micelle structures to near-cylindrical ensembles (worm-like micelles). On the other hand, the micelle size and shape of the core-shell spherical micelles adopted by Pluronic F127 is independent of the temperature, with the arrangement of these nanodomains into a macrocrystalline network leading to the formation of an elastic gel. The gel structure was visualized via cryoTEM and cryoSEM. This novel methacrylate polymer exhibited excellent biocompatibility and great potential as an injectable intracameral formulation for ocular drug delivery.

## Supporting Information Available

(Figures S1–S20) Additional rheological measurements; gel permeation chromatograms (GPC); nuclear magnetic resonance (NMR) spectra; injection ratios; photographs of gels; evolution of storage modulus with concentration; complementary differential scanning thermograms; complementary phase diagrams; complementary small-angle neutron scattering data; additional image by cryogenic transmission electron microscopy; additional images on in vitro and ex vivo gelation; drug loading content and encapsulation efficiency as a function of time; (Tables S1 and S2) GPC and NMR results; dynamic light scattering (DLS) data; additional small-angle neutron scattering (SANS) data (PDF)

## Notes

A.P.C. and T.K.G. report that part of this work regarding the novel chemistry of the materials and their promising thermoresponsive properties have been patented.^100^

## Notes

Research Square was used to prepare one of the earliest versions of the manuscript: DOI: 10.21203/rs.3.rs-104434/v1.

## ABBREVIATIONS

APRE-19: arising retinal pigment epithelia
b.t.: body temperature
BuMA: *n*-butyl methacrylate
CP: cloud point
CGC: critical gelation concentrations
CGT: critical gelation temperature
cryoTEM: cryogenic transmission microscopy
D_2_O/PBS: deuterated phosphate-buffered saline
DEGMA: di(ethylene glycol) methyl ether methacrylate
DSC: differential scanning calorimetry
3D: three-dimensional
DMSO: dimethylsulfoxide
DPPH: 2-diphenyl-1-picrylhydrazyl hydrate
DMEM: Dulbecco’s modified Eagle medium
DLS: dynamic light scattering
EG: ethylene glycol
Δ*H*_micell_: enthalpy of micellization
*T*_gel_: gelation temperature
GPC: gel permeation chromatography
GTP: group transfer polymerization
LCST: lower critical solution temperature
MTS: methyl trimethylsilyl dimethylketene acetal
MM: molecular mass
NMR: nuclear magnetic resonance
OEGMA300: oligo(ethylene glycol) methyl ether methacrylate with *M*_n_ = 300 g mol^–1^
*T*_onset_: onset temperature
PBS: phosphate-buffered saline
PLGA: poly(d,l-lactide-*co*-glycolide)
PEI: polyethyleneimine
PMMA: poly(methyl methacrylate)
PTFE: polytetrafluoroethylene
PG: propylene glycol
Rheo-SANS: rheological small-angle neutron scattering
r.t.: room temperature
cryoSEM: scanning electron microscopy
SF: sodium fluorescein
TBABB: tetrabutylammonium bibenzoate
THF: tetrahydrofuran
TRG: thermoreversible hydrogels
TE: tissue engineering
UV–Vis: ultraviolet–visible
UK: United Kingdom
