# Supplementary material for: Investigation of the Thermogelation of a Promising Biocompatible ABC Triblock Terpolymer and Its Comparison with Pluronic F127
Source: Macromolecules. 2022 Feb 14;55(5):1783–99. doi: 10.1021/acs.macromol.1c02123 (PMC9007541; doi:10.1021/acs.macromol.1c02123)
Supplement: Supplementary file 1 — ma1c02123_si_001.pdf [file ma1c02123_si_001.pdf]

Investigation of the thermogelation of a promising biocompatible ABC triblock terpolymer and its comparison with Pluronic® F127

Anna P. Constantinou,[a] Valeria Nele,[a,b,c] James J. Doutch,[d] Joana S. Correia,[a] Roman V. Moiseev,[e] Martina Cihova,[a,b,c] David C. A. Gaboriau,[f] Jonathan Krell,[g] Vitaliy V. Khutoryanskiy,[e] Molly M. Stevens[a,b,c] and Theoni K. Georgiou*[a]

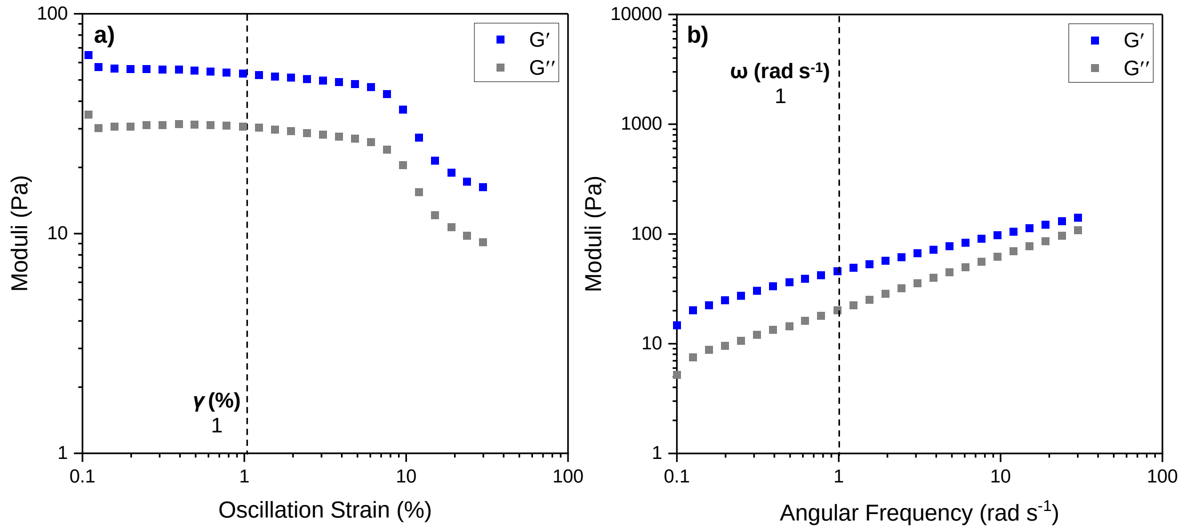

Figure S1: Rheological measurements on OEGMA300$_{15}$-*b*-BuMA$_{26}$-*b*-DEGMA$_{13}$ in deuterated phosphate buffered saline (D_2O/PBS) at 15 w/w% at 37°C: (a) amplitude-sweep measurement – storage modulus, G′, in blue, and loss modulus, G″, in grey, and (b) frequency-sweep measurement – storage modulus, G′, in blue, and loss modulus, G″, in grey.

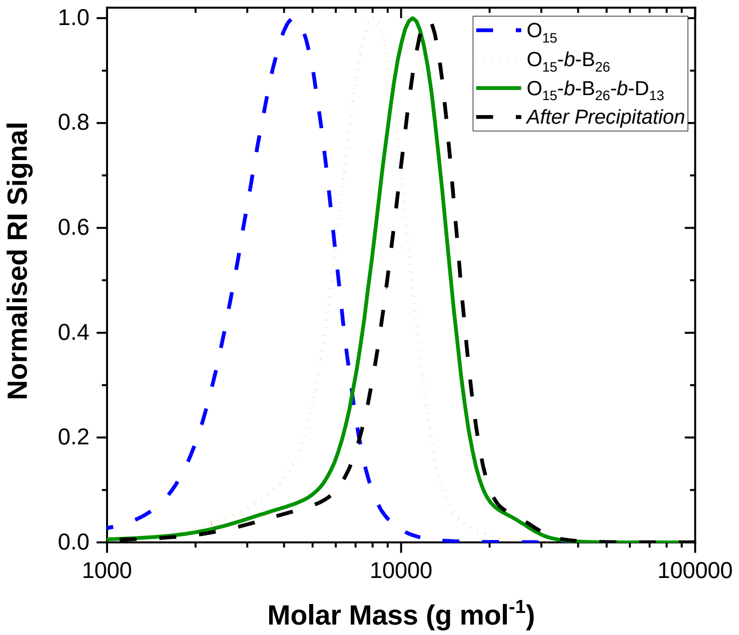

Figure S2. Normalized GPC traces proving the successful synthesis of $OEGMA300_{15}$-*b*-$BuMA_{16}$-*b*-$DEGMA_{13}$: i) $OEGMA300_{15}$ homopolymer in blue dashed line, ii) $OEGMA300_{15}$-*b*-$BuMA_{26}$ diblock copolymer in orange dotted line, iii) $OEGMA300_{15}$-*b*-$BuMA_{26}$-*b*-$DEGMA_{13}$ triblock terpolymer before precipitation in green solid line, and iv) the final triblock terpolymer after precipitation in black dashed line, respectively.

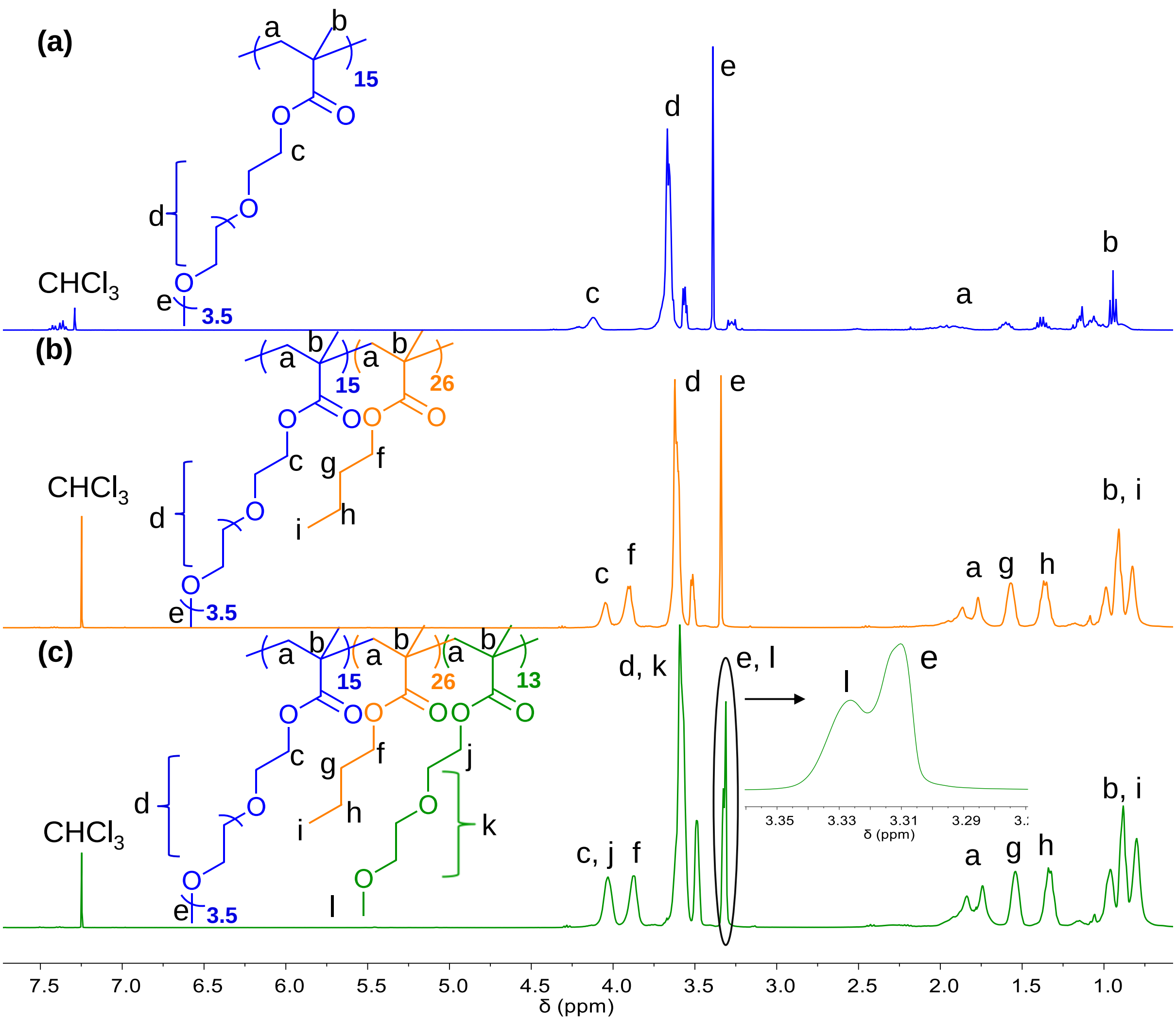

Figure S3. ^{1}H NMR spectra of OEGMA300$_{15}$-*b*-BuMA$_{26}$-*b*-DEGMA$_{13}$ and its linear precursors: (a) OEGMA300$_{15}$ homopolymer in blue, (b) OEGMA300$_{15}$-*b*-BuMA$_{26}$ diblock copolymer in orange, and (c) OEGMA300$_{15}$-*b*-BuMA$_{26}$-*b*-DEGMA$_{13}$ triblock terpolymer in green, respectively. The polymer structures of each step are also presented, in which the OEGMA300, BuMA, and DEGMA units are coloured in blue, orange and green, respectively.

Table S1: Theoretical polymer structure, molar mass (MM) and composition, and experimental molar mass/ number-average molar mass (M_n), molar mass distributions ($Đ$) and compositions.

Theoretical Structure [a]	$MM^{theor. b}$ (g mol^{-1})	M_n [c] (g mol^{-1})	$Đ$ [c]	OEGMA300-BuMA-DEGMA (w/w%)		Experimental Structure [a]
				Theoretical	^{1}H NMR	
$OEGMA300_{10}$	3200	3560	1.16	100-00-00	100-00-00	$OEGMA300_{15}$
$OEGMA300_{10}$-*b*-$BuMA_{18}$	5725	6920	1.14	53-47-00	55-45-00	$OEGMA300_{15}$-*b*-$BuMA_{26}$
$OEGMA300_{10}$-*b*-$BuMA_{18}$-*b*-$DEGMA_{10}$	7600	9100<br>10400 *	1.18<br>1.15 *	40-35-25	42-35-23	$OEGMA300_{15}$-*b*-$BuMA_{26}$-*b*-$DEGMA_{13}$

[a] OEGMA300, BuMA, DEGMA and PMA are the abbreviations for oligo(ethylene glycol) methyl ether methacrylate with M_n 300 g mol^{-1}, *n*-butyl methacrylate, and di(ethylene glycol) methyl ether methacrylate, respectively.

[b] The theoretical MM has been calculated by the following equation: $MMtheor.(g * mol^{-1}) = \sum_0^n (MM^{repeated\ unit} * DP) + 100$; where DP stands for degree of polymerisation and 100 g mol^{-1} is the MM of the part of the initiator that stays on the polymer chain.

[c] The number-average MM (M_n) and the dispersity have resulted by gel permeation chromatography (GPC) analysis. The calibration is based on six MMA standard samples of the following MM values: 2, 4, 8, 20, 50, 100 kg mol^{-1}.

* The results correspond to the GPC analysis after precipitation.

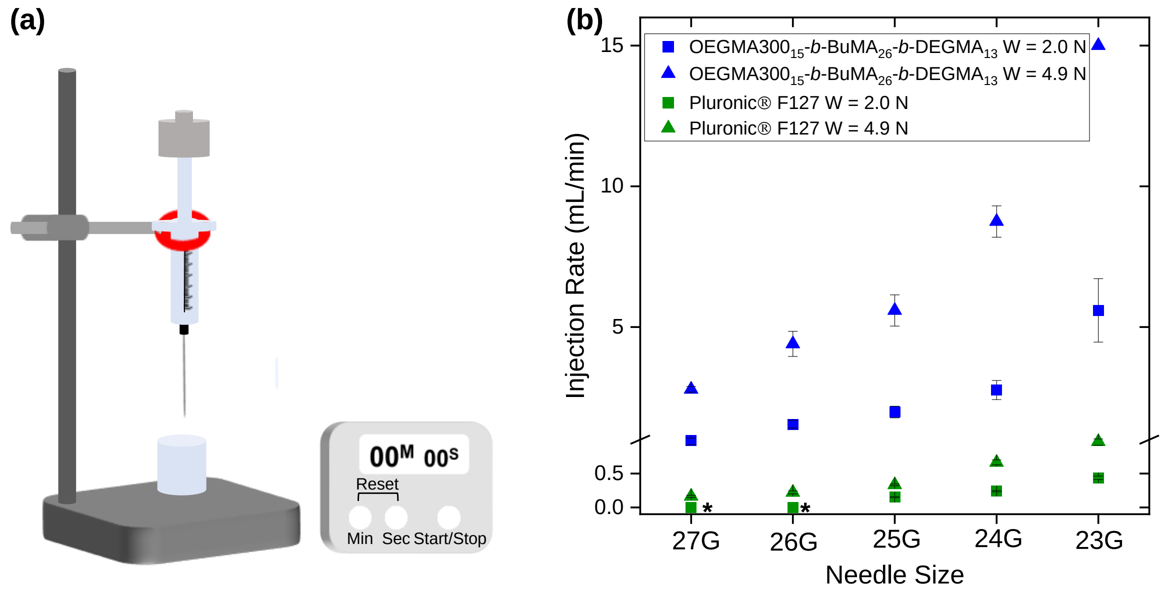

* The injection rate is equal to 0 mL/min, as the solution did not pass through the needle at the specific force applied (W = 2.0 N).

Figure S4. a) Experimental setup for measuring the injection rate and b) dependence of the injection rate (mL/min) of the solutions of OEGMA300$_{15}$-*b*-BuMA$_{26}$-*b*-DEGMA$_{13}$ (in blue) and Pluronic® F127 (in green) at 15 w/w% in PBS on the size of the needle. The injection rate was measured by using two different weights: i) 200 g (2 N) shown in squares and ii) 500 g (4.9 N) shown in triangles.

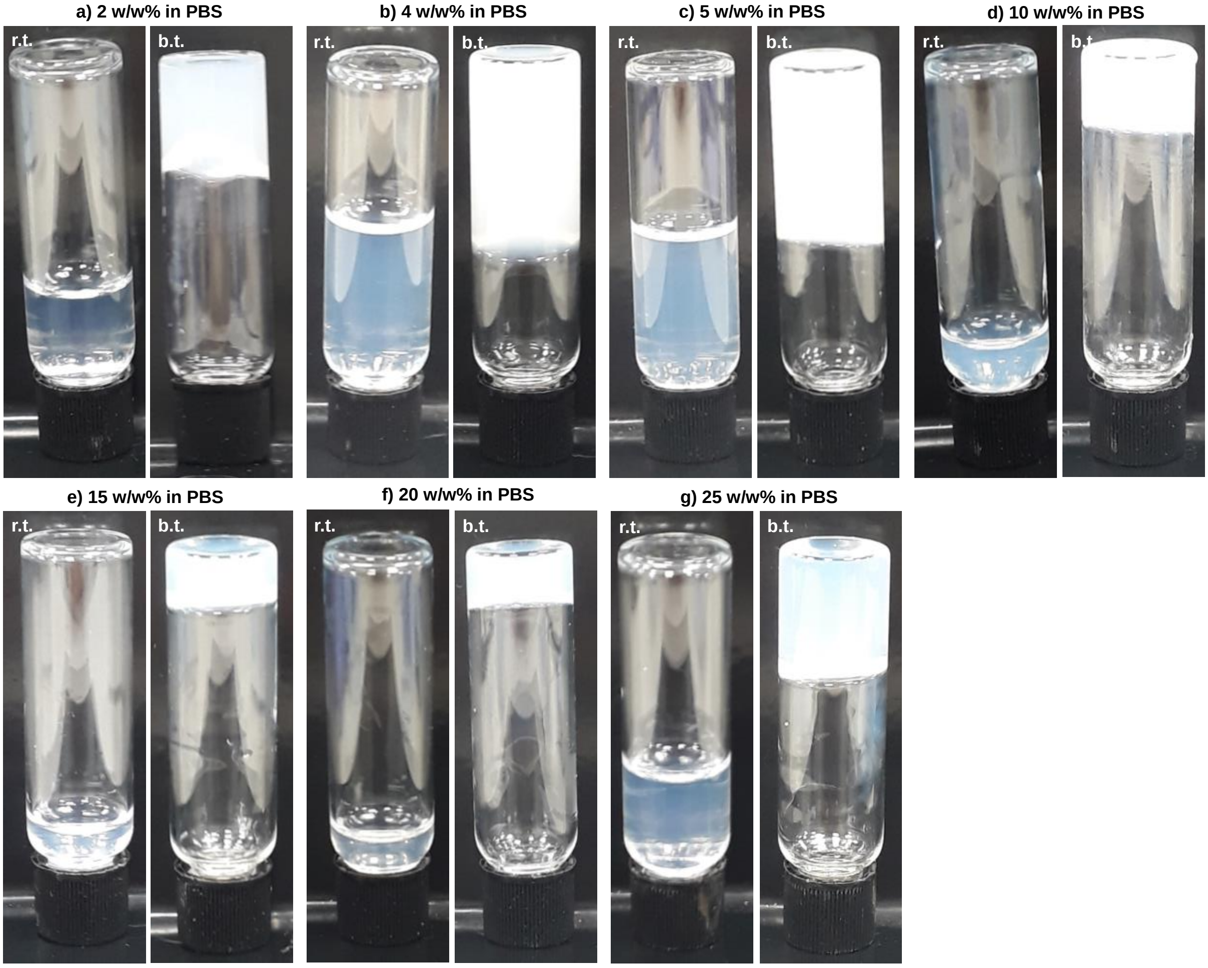

Figure S5: Photographs of the solutions of $OEGMA300_{15}$-*b*-$BuMA_{26}$-*b*-$DEGMA_{13}$ at various concentrations in phosphate buffered saline (PBS) at the solution state (at room temperature, r.t.) and at the gel phase (at body temperature, b.t.).

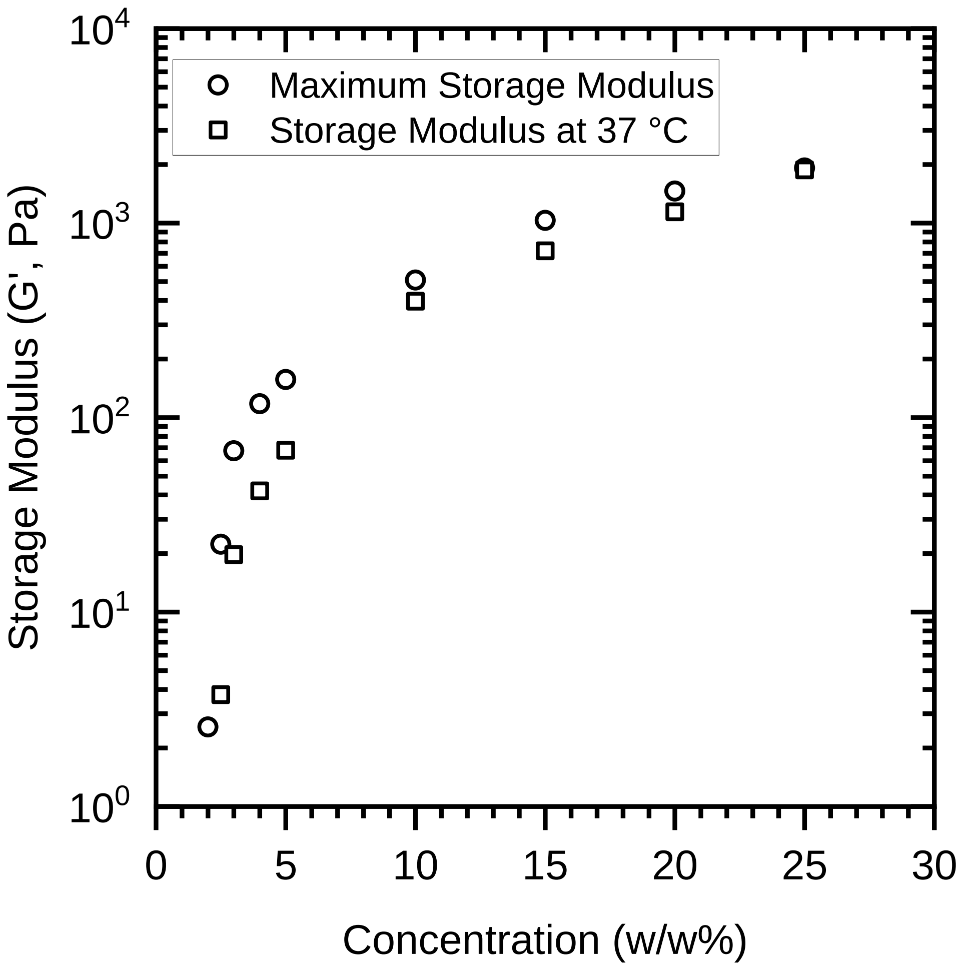

Figure S6: Storage modulus (G′, Pa) as a function of the polymer concentration in phosphate buffered saline (PBS) for OEGMA300$_{15}$-*b*-BuMA$_{26}$-*b*-DEGMA$_{13}$. The maximum storage modulus is indicated by circles, while the storage modulus at 37 °C is shown in squares.

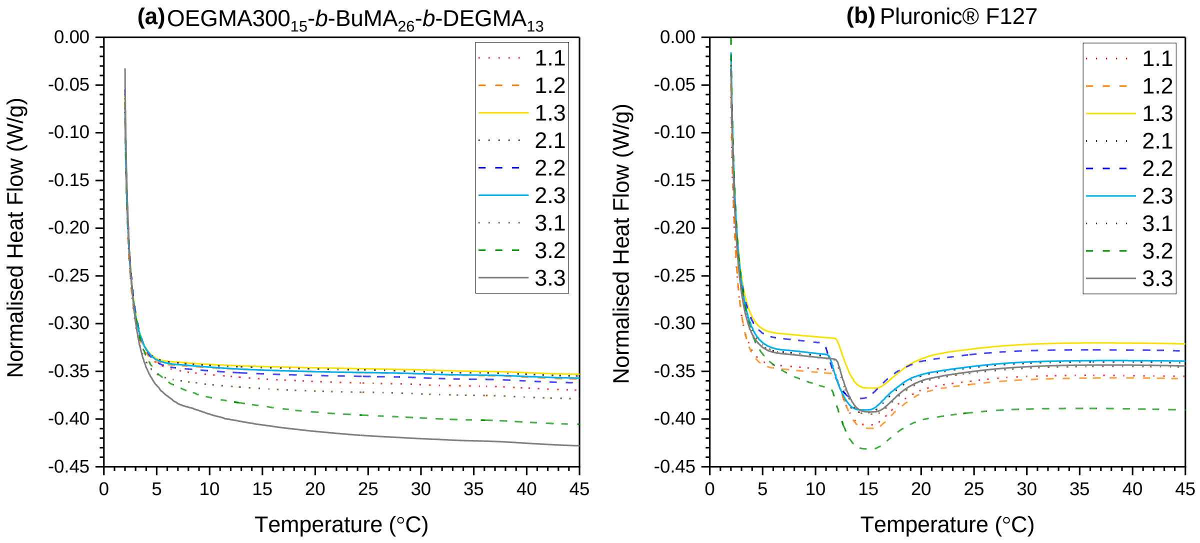

Figure S7. DSC thermograms of 15 w/w% polymer solutions in phosphate buffered saline (PBS); (a) $OEGMA300_{15}$-*b*-$BuMA_{26}$-*b*-$DEGMA_{13}$ and (b) Pluronic® F127. The first, second, and third runs of each batch of polymer solution are shown in dotted, dashed and solid lines, respectively.

Table S2: Polymer structure, hydrodynamic diameters (d_H) and polydispersity indices as resulted by dynamic light scattering (DLS) in phosphate buffered saline (PBS) at 10°C and 25°C.

**Polymer Structure** [a]	**Hydrodynamic Diameter (d_H, nm)**					
	**Temp. (°C)**	**By Intensity** [b]			**By Number** [b]	**PDI**
		**1st**	**2nd**	**3rd**		
OEGMA300$_{15}$-*b*-BuMA$_{26}$-*b*-DEGMA$_{13}$	10	----	37.8	----	15.7	0.219
	25	----	43.8	----	21.0	0.207
EG$_{99}$-*b*-PG$_{66}$-*b*-EG$_{99}$	10	**8.8**	----	342.0	6.5	0.144
	25	6.5	**28.2**	----	5.6	0.276

[a] OEGMA300, BuMA, and DEGMA stand for oligo(ethylene glycol) methyl ether methacrylate with M_n 300 g mol^{-1}, *n*-butyl methacrylate, and di(ethylene glycol) methyl ether methacrylate, respectively. EG and PG stand for ethylene glycol and propylene glycol, respectively.

[b] The results presented are the diameter which corresponds to the maximum of the peak by intensity and by number; where more than one peaks are present, the value of highest intensity is shown in bold.

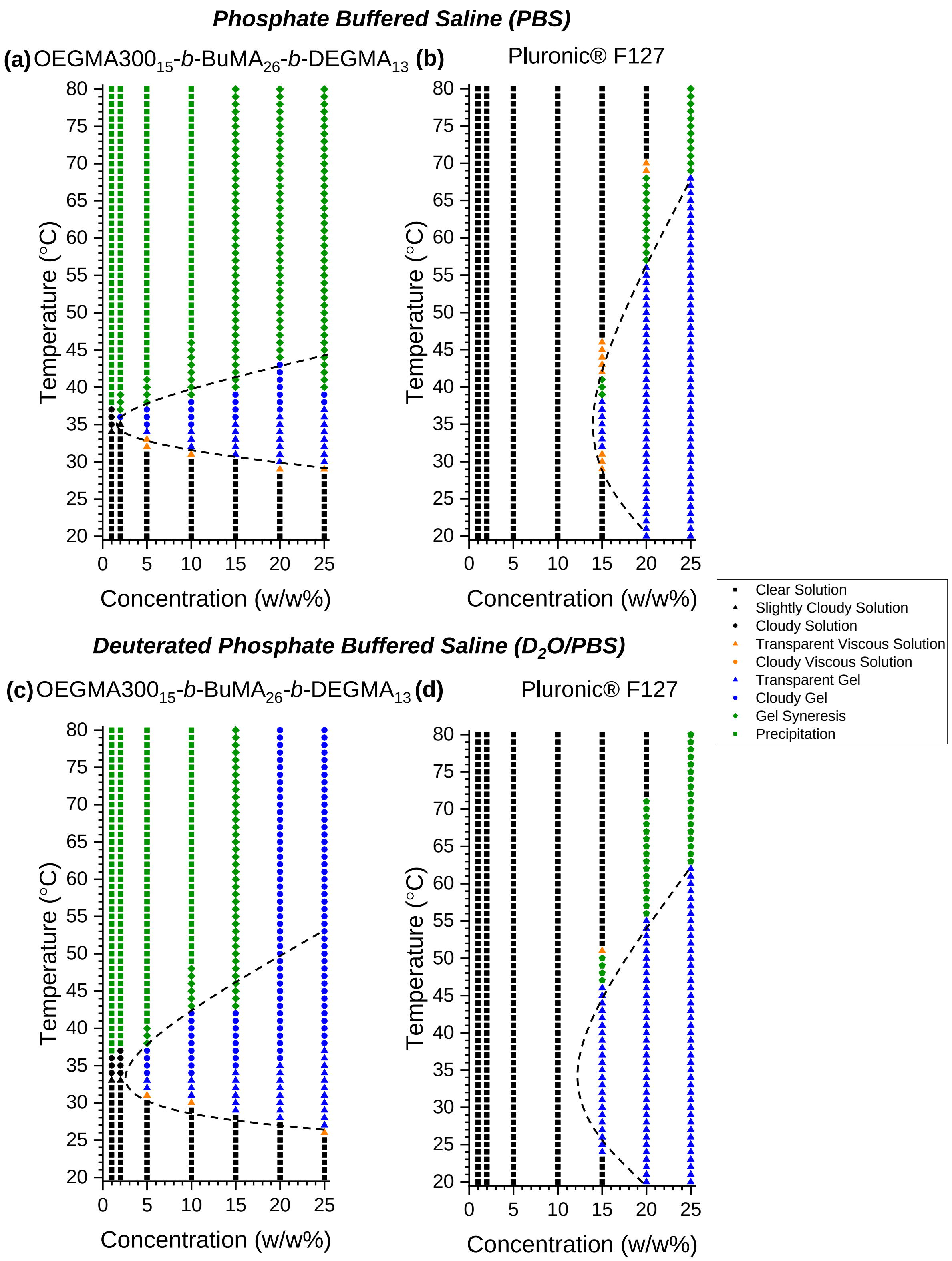

Figure S8. Detailed phase diagrams in phosphate buffered saline (PBS, top), and deuterated phosphate buffered saline (D_2O/PBS, bottom) for the samples of OEGMA300$_{15}$-*b*-BuMA$_{26}$-*b*-DEGMA$_{13}$ (a) and (c) and Pluronic® F127 (b) and (d). The gelation area is approximately shown in black dashed line.

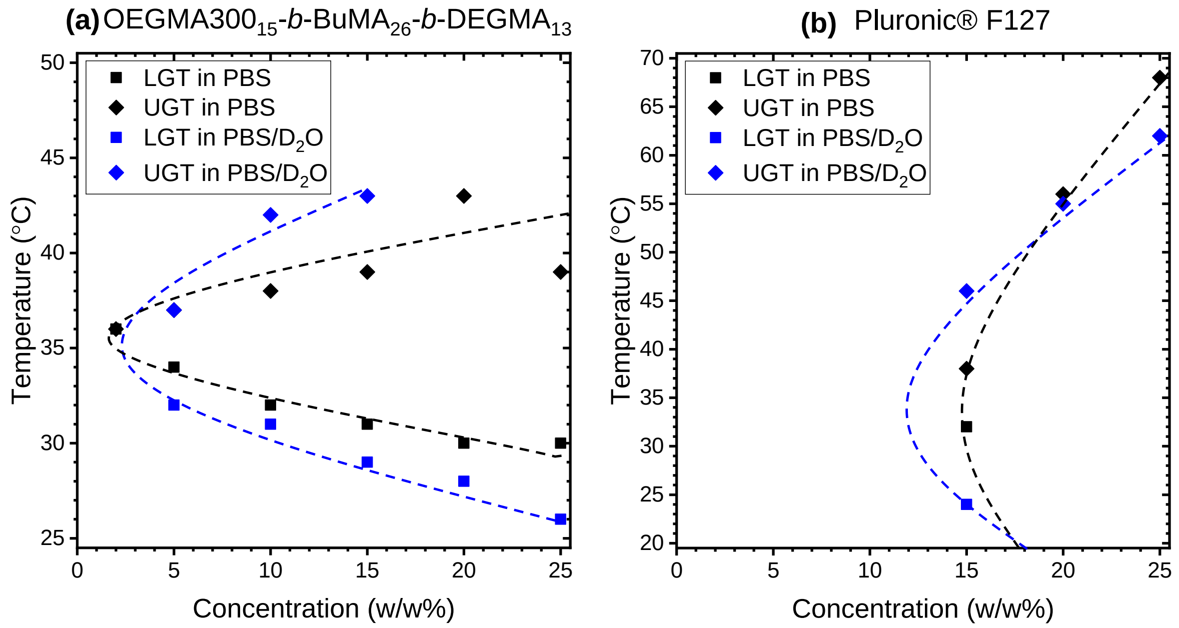

Figure S9. Phase diagrams indicating the gelation boundaries in phosphate buffered saline (PBS, black square for lower gelation temperature, LGT, and black rhombus for upper gelation temperature, UGT) and in deuterated phosphate buffered saline (D_2O/PBS, blue square for LGT, and blue rhombus for UGT) for OEGMA300$_{15}$-b-BuMA$_{26}$-b-DEGMA$_{13}$ (left) and Pluronic® F127 (right). The gelation areas are approximately shown in dashed lines (black for PBS, and blue for D_2O/PBS).

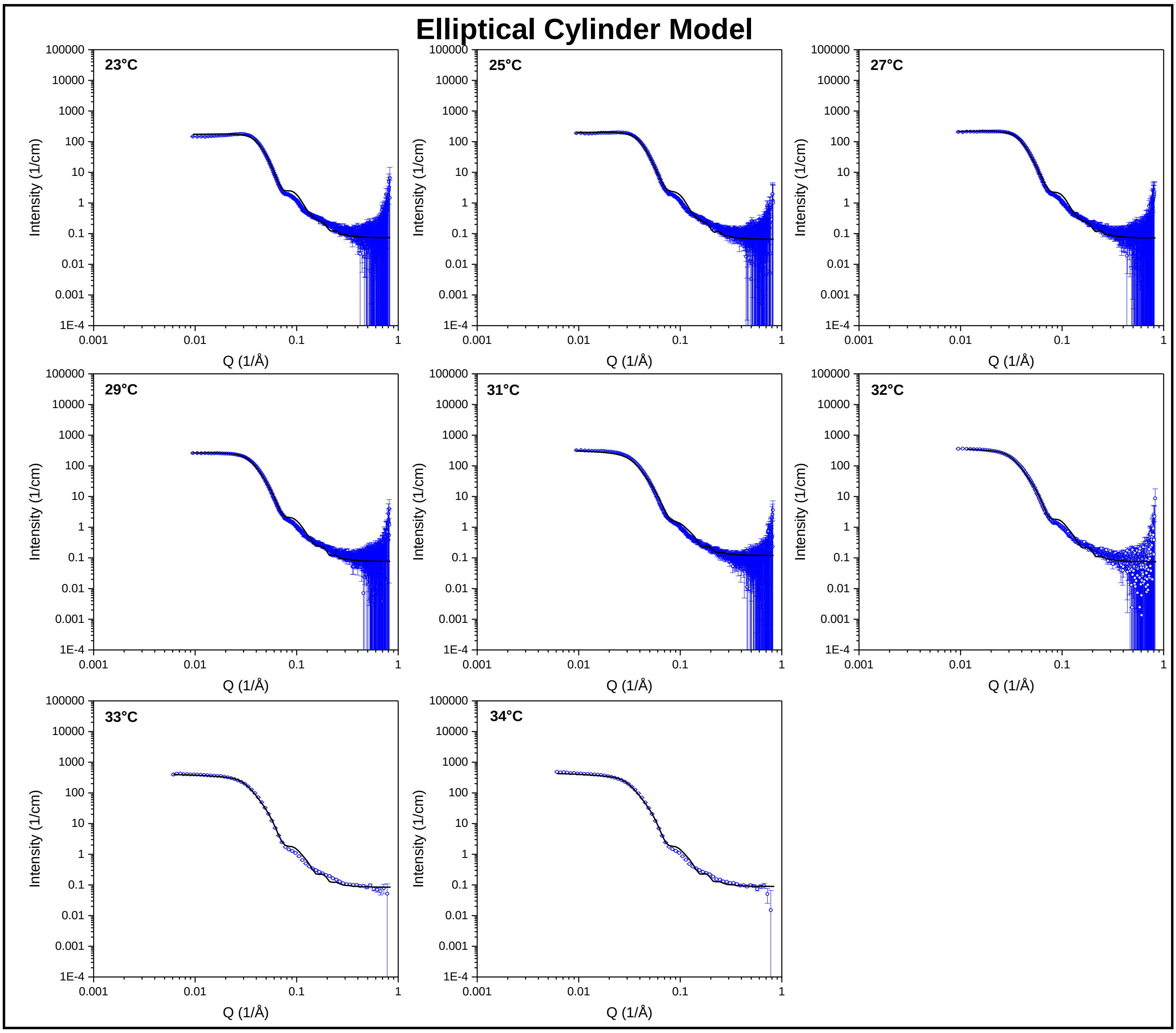

Figure S10. SANS data from the solutions of OEGMA300$_{15}$-*b*-BuMA$_{26}$-*b*-DEGMA$_{13}$ at 15 w/w% in deuterated phosphate buffered saline (D_2O/PBS) at different temperatures (23°C to 34°C). The data points along with their errors are presented in blue, whereas the fit lines generated by using an Elliptical Cylinder model with hardsphere, with the polydispersity of the length being fixed at 0.15, in SasView software, are shown in black.

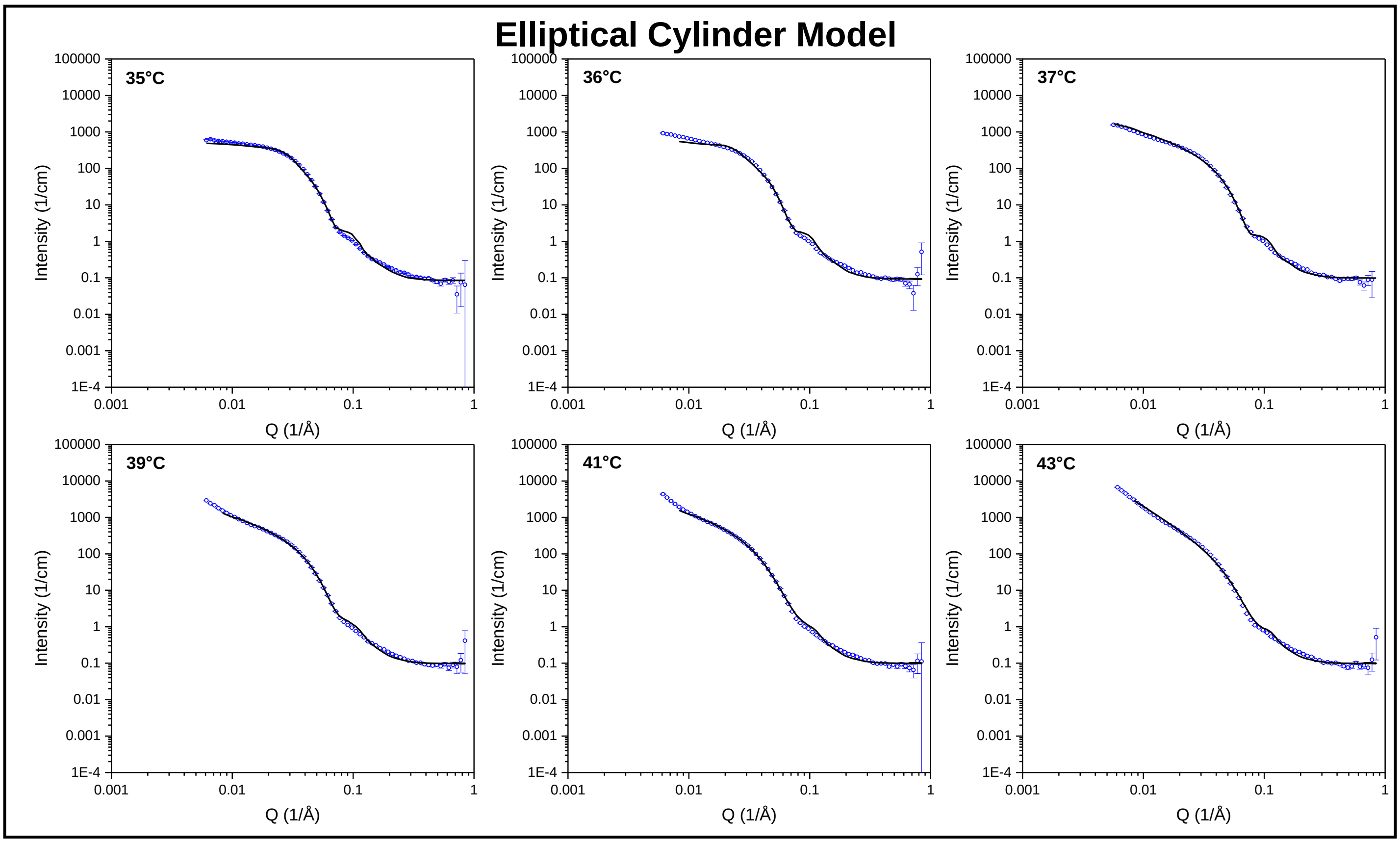

Figure S11. SANS data from the solutions of OEGMA300$_{15}$-*b*-BuMA$_{26}$-*b*-DEGMA$_{13}$ at 15 w/w% in deuterated phosphate buffered saline (D_2O/PBS) at different temperatures (35°C to 43°C). The data points along with their errors are presented in blue, whereas the fit lines generated by using an Elliptical Cylinder model with hardsphere, with the polydispersity of the radius minor being fixed at 0.15 in SasView software, are shown in black.

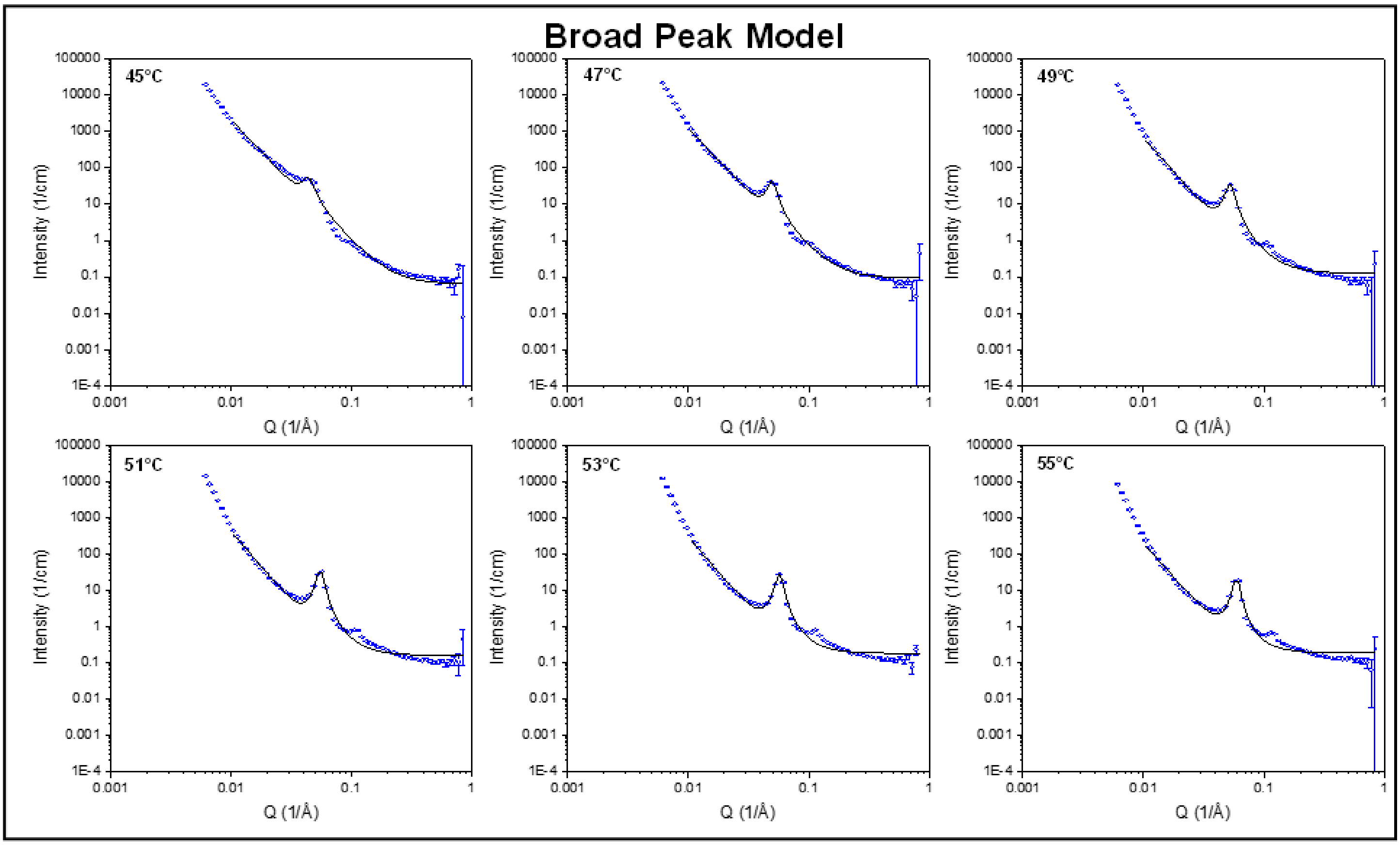

Figure S12. SANS data from the solutions of OEGMA300$_{15}$-*b*-BuMA$_{26}$-*b*-DEGMA$_{13}$ at 15 w/w% in deuterated phosphate buffered saline (D_2O/PBS) at different temperatures (45°C to 55°C). The data points along with their errors are presented in blue, whereas the fit lines, generated by using a Broad Peak model in SasView software, are shown in black.

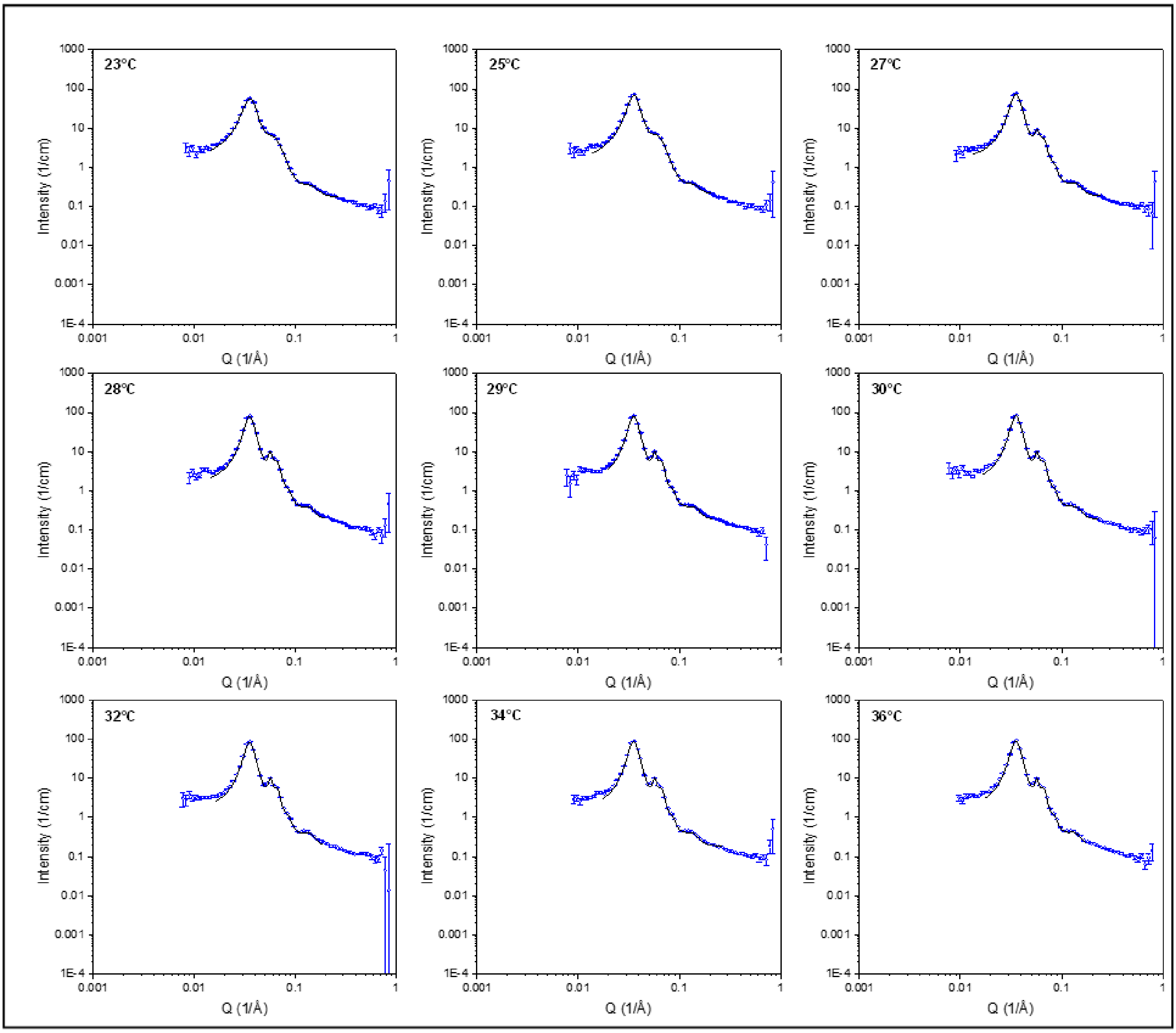

Figure S13. SANS data from the solutions of Pluronic® F127 at 15 w/w% in deuterated phosphate buffered saline (D_2O/PBS) at different temperatures (23°C to 36°C). The data points along with their errors are presented in blue, whereas the fit lines, generated by using the small-angle diffraction tool in IRENA in IGOR software, are shown in black.

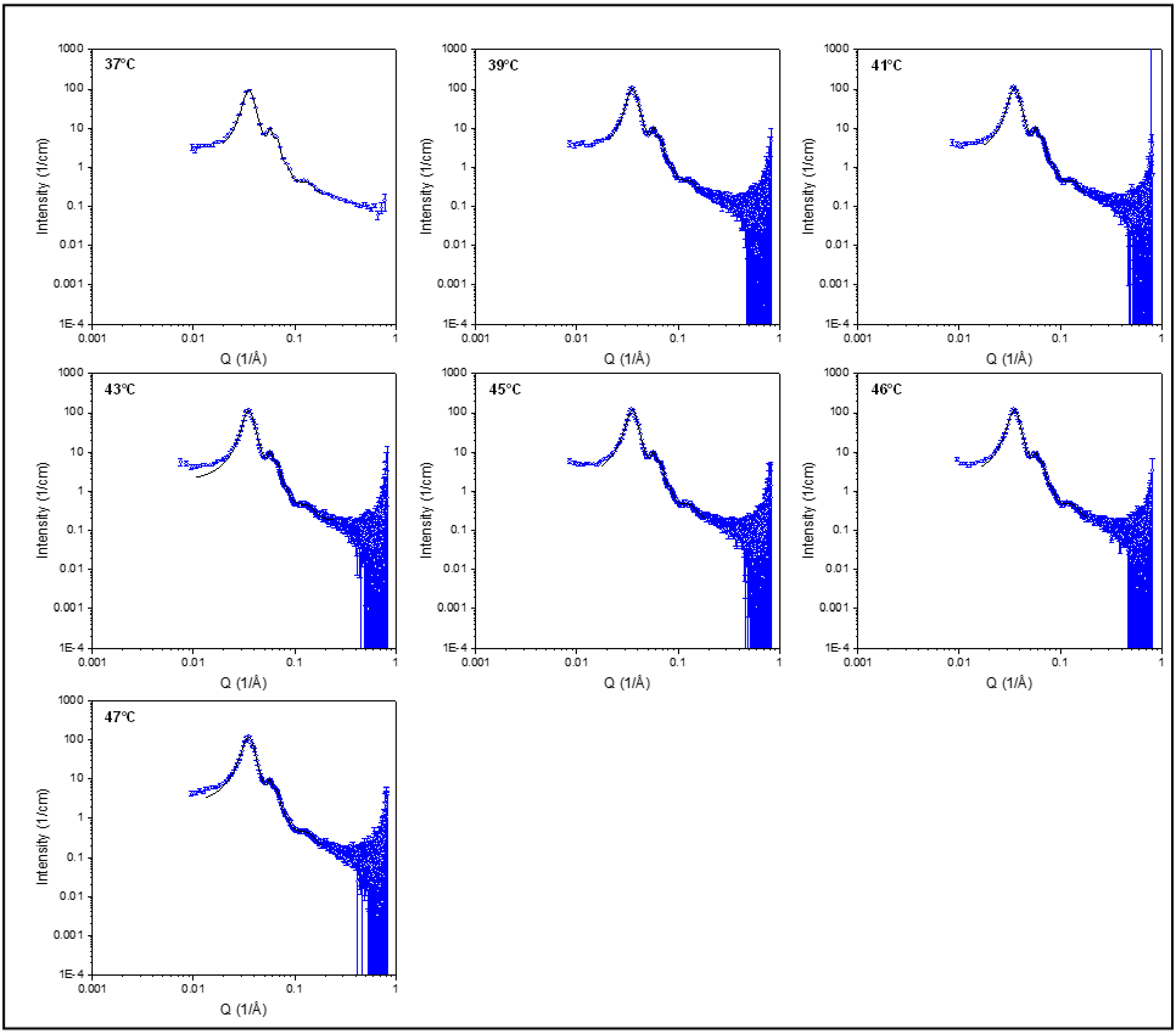

Figure S14. SANS data from the solutions of Pluronic® F127 at 15 w/w% in deuterated phosphate buffered saline (D_2O/PBS) at different temperatures (39°C to 47°C). The data points along with their errors are presented in blue, whereas the fit lines, generated by using the small-angle diffraction tool in IRENA in IGOR software, are shown in black.

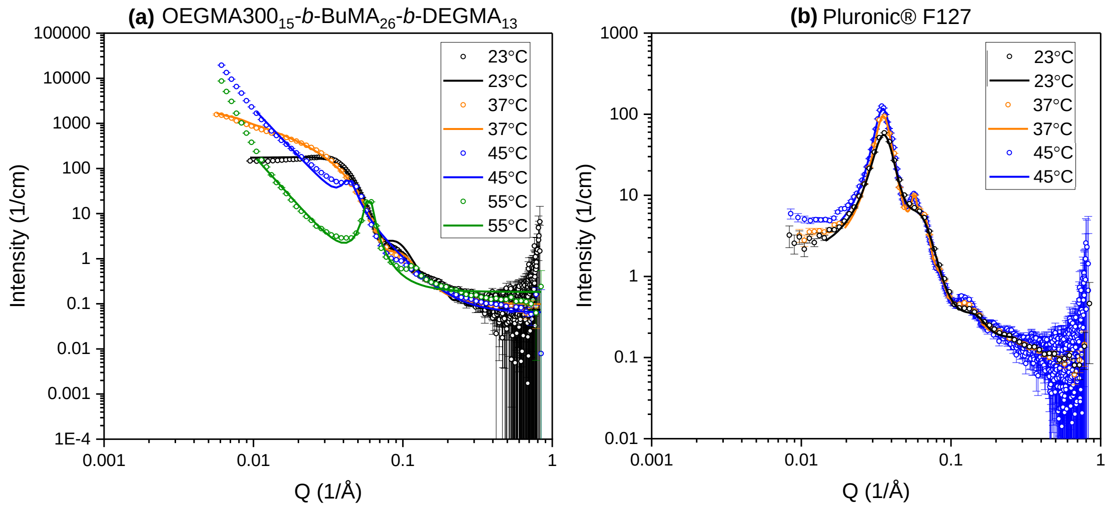

Figure S15. SANS data from 15 w/w% solutions in deuterated phosphate buffered saline (D_2O/PBS) of (a) OEGMA300$_{15}$-*b*-BuMA$_{26}$-*b*-DEGMA$_{13}$ and (b) Pluronic® F127, at different temperatures. The data points along with their errors, and their fit lines at different temperatures are presented in: i) black for 23°C (starting temperature), ii) orange for 37°C (body temperature), iii) blue for 45°C, and iv) green for 55°C for our polymer only. The fit lines were generated by using: i) an Elliptical Cylinder model with hardsphere in SasView software for OEGMA300$_{15}$-*b*-BuMA$_{26}$-*b*-DEGMA$_{13}$ at 23°C and 37°C, ii) a Broad Peak model in SasView software for OEGMA300$_x$-*b*-BuMA$_y$-*b*-DEGMA$_z$ at 45°C and 55°C, iii) the small-angle diffraction tool in IRENA in IGOR software for Pluronic® F127 at 23°C, 37°C, and 45°C.

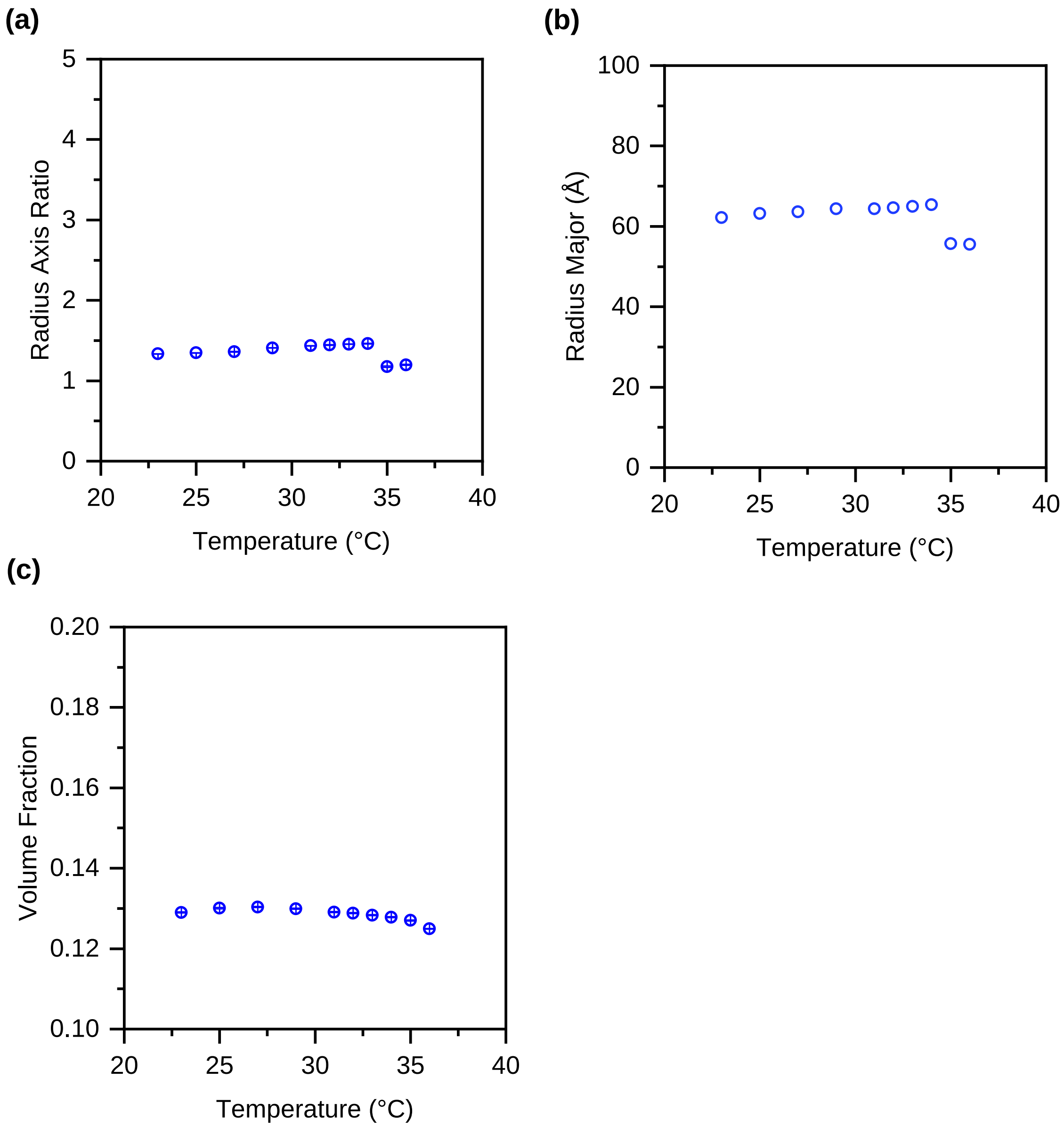

Figure S16. The additional fitting parameters of the self-assembled structure adopted by OEGMA300$_{15}$-*b*-BuMA$_{26}$-*b*-DEGMA$_{13}$ as a function of temperature are presented: (a) radius axis ratio, (b) radius major, calculated as the product of the radius minor and the axis ratio, and (c) volume fraction.

Table S3: Peak positions identified in the SANS patterns resulted from 15 w/w% Pluronic® F127 solutions in deuterated phosphate buffered saline (D_2O/PBS). The small-angle diffraction tool in IRENA in IGOR software was used to fit the data.

Temperature (°C)	Peak Position									
	(Q, Å^{-1})					d (Å)				
	1st	2nd	3rd	4th	5th	1st	2nd	3rd	4th	5th
23	0.0355	0.0615	–	–	0.1515	176.99	102.17	–	–	41.473
25	0.0355	0.0605	–	–	0.1435	176.99	103.85	–	–	43.785
27	0.0355	0.0575	0.0665	0.0855	0.1465	176.99	109.27	94.484	73.488	42.889
28	0.0355	0.0565	0.0665	0.0855	0.1425	176.99	111.21	94.484	73.488	44.093
29	0.0355	0.0565	0.0665	0.0855	0.1455	176.99	111.21	94.484	73.488	43.183
30	0.0355	0.0575	0.0665	0.0845	0.1435	176.99	109.27	94.484	74.357	43.785
32	0.0355	0.0565	0.0665	0.0855	0.1435	176.99	111.21	94.484	73.488	43.785
34	0.0355	0.0565	0.0665	0.0845	0.1415	176.99	111.21	94.484	74.357	44.404
36	0.0355	0.0565	0.0665	0.0855	0.1405	176.99	111.21	94.484	73.488	44.72
37	0.0355	0.0565	0.0665	0.0845	0.1415	176.99	111.21	94.484	74.357	44.404
39	0.0355	0.0575	0.0665	0.0835	0.1405	176.99	109.27	94.484	75.248	44.72
41	0.0345	0.0575	0.0665	0.0855	0.1395	182.12	109.27	94.484	73.488	45.041
43	0.0345	0.0575	0.0665	0.0845	0.1435	182.12	109.27	94.484	74.357	43.785
45	0.0345	0.0565	0.0665	0.0845	0.1385	182.12	111.21	94.484	74.357	45.366
46	0.0345	0.0565	0.0665	0.0835	0.1405	182.12	111.21	94.484	75.248	44.72
47	0.0345	0.0565	0.0665	0.0835	0.1405	182.12	111.21	94.484	75.248	44.72

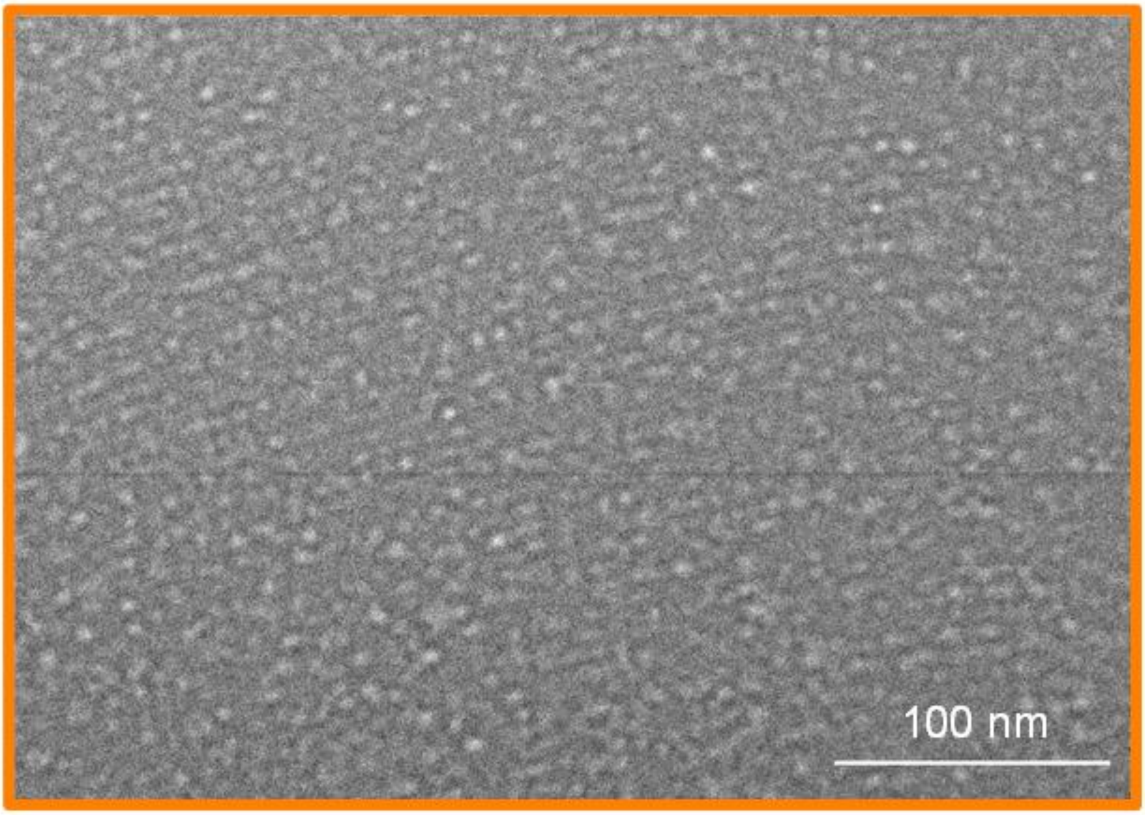

Figure S17. CryoTEM image of the Pluronic® F127 gel at 37 °C, which shows the organised orientation of the micelles.

(a) OEGMA300$_{15}$-*b*-BuMA$_{26}$-*b*-DEGMA$_{13}$ (b) Pluronic® F127

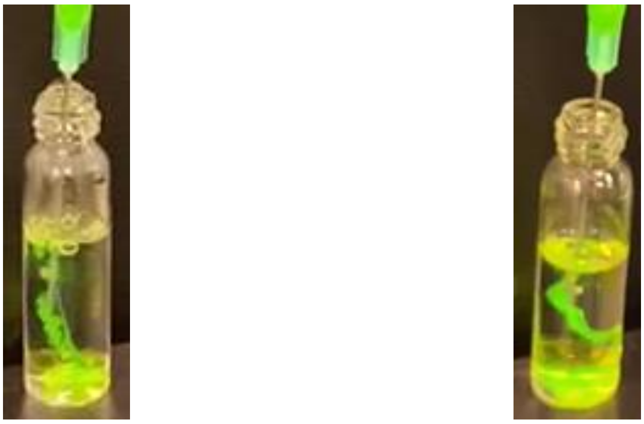

Figure S18. Images of *in vitro* gelation of 15 w/w% solutions in phosphate buffered saline (PBS) upon injection in PBS at 37 °C: (a) OEGMA300$_{15}$-*b*-BuMA$_{26}$-*b*-DEGMA$_{13}$ and (b) Pluronic® F127.

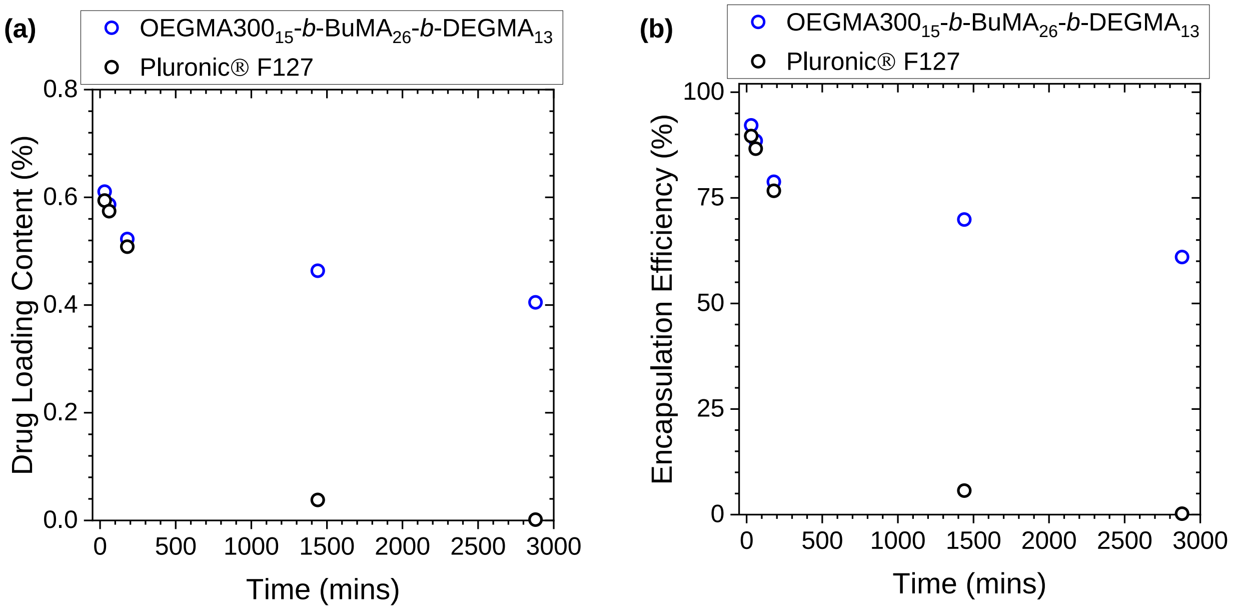

Figure S19. (a) Drug loading content (%) and (b) encapsulation efficiency (%) of the gels formed by OEGMA300$_{15}$-*b*-BuMA$_{26}$-*b*-DEGMA$_{13}$ (blue) and (b) Pluronic® F127 (black). Sodium fluorescein was used as a model drug.

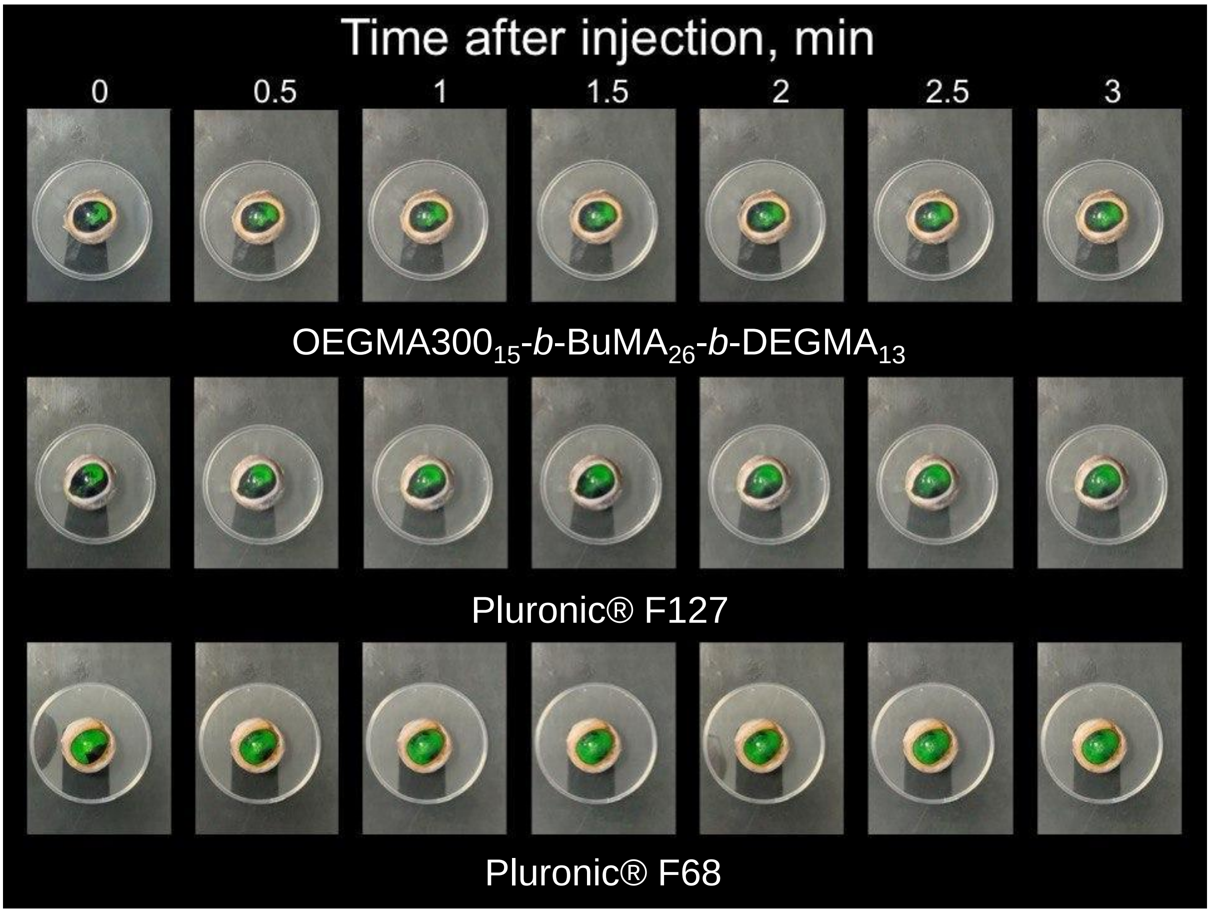

Figure S20. *Ex vivo* intracameral injections of 15 w/w % solutions in PBS, containing 1 mg/mL sodium fluorescein, into bovine eyes.
